# Impact of common genetic determinants of Hemoglobin A1c on type 2 diabetes risk and diagnosis in ancestrally diverse populations: A transethnic genome-wide meta-analysis

**DOI:** 10.1371/journal.pmed.1002383

**Published:** 2017-09-12

**Authors:** Eleanor Wheeler, Aaron Leong, Ching-Ti Liu, Marie-France Hivert, Rona J. Strawbridge, Clara Podmore, Man Li, Jie Yao, Xueling Sim, Jaeyoung Hong, Audrey Y. Chu, Weihua Zhang, Xu Wang, Peng Chen, Nisa M. Maruthur, Bianca C. Porneala, Stephen J. Sharp, Yucheng Jia, Edmond K. Kabagambe, Li-Ching Chang, Wei-Min Chen, Cathy E. Elks, Daniel S. Evans, Qiao Fan, Franco Giulianini, Min Jin Go, Jouke-Jan Hottenga, Yao Hu, Anne U. Jackson, Stavroula Kanoni, Young Jin Kim, Marcus E. Kleber, Claes Ladenvall, Cecile Lecoeur, Sing-Hui Lim, Yingchang Lu, Anubha Mahajan, Carola Marzi, Mike A. Nalls, Pau Navarro, Ilja M. Nolte, Lynda M. Rose, Denis V. Rybin, Serena Sanna, Yuan Shi, Daniel O. Stram, Fumihiko Takeuchi, Shu Pei Tan, Peter J. van der Most, Jana V. Van Vliet-Ostaptchouk, Andrew Wong, Loic Yengo, Wanting Zhao, Anuj Goel, Maria Teresa Martinez Larrad, Dörte Radke, Perttu Salo, Toshiko Tanaka, Erik P. A. van Iperen, Goncalo Abecasis, Saima Afaq, Behrooz Z. Alizadeh, Alain G. Bertoni, Amelie Bonnefond, Yvonne Böttcher, Erwin P. Bottinger, Harry Campbell, Olga D. Carlson, Chien-Hsiun Chen, Yoon Shin Cho, W. Timothy Garvey, Christian Gieger, Mark O. Goodarzi, Harald Grallert, Anders Hamsten, Catharina A. Hartman, Christian Herder, Chao Agnes Hsiung, Jie Huang, Michiya Igase, Masato Isono, Tomohiro Katsuya, Chiea-Chuen Khor, Wieland Kiess, Katsuhiko Kohara, Peter Kovacs, Juyoung Lee, Wen-Jane Lee, Benjamin Lehne, Huaixing Li, Jianjun Liu, Stephane Lobbens, Jian'an Luan, Valeriya Lyssenko, Thomas Meitinger, Tetsuro Miki, Iva Miljkovic, Sanghoon Moon, Antonella Mulas, Gabriele Müller, Martina Müller-Nurasyid, Ramaiah Nagaraja, Matthias Nauck, James S. Pankow, Ozren Polasek, Inga Prokopenko, Paula S. Ramos, Laura Rasmussen-Torvik, Wolfgang Rathmann, Stephen S. Rich, Neil R. Robertson, Michael Roden, Ronan Roussel, Igor Rudan, Robert A. Scott, William R. Scott, Bengt Sennblad, David S. Siscovick, Konstantin Strauch, Liang Sun, Morris Swertz, Salman M. Tajuddin, Kent D. Taylor, Yik-Ying Teo, Yih Chung Tham, Anke Tönjes, Nicholas J. Wareham, Gonneke Willemsen, Tom Wilsgaard, Aroon D. Hingorani, EPIC-CVD Consortium, EPIC-InterAct Consortium, Lifelines Cohort Study, Josephine Egan, Luigi Ferrucci, G. Kees Hovingh, Antti Jula, Mika Kivimaki, Meena Kumari, Inger Njølstad, Colin N. A. Palmer, Manuel Serrano Ríos, Michael Stumvoll, Hugh Watkins, Tin Aung, Matthias Blüher, Michael Boehnke, Dorret I. Boomsma, Stefan R. Bornstein, John C. Chambers, Daniel I. Chasman, Yii-Der Ida Chen, Yduan-Tsong Chen, Ching-Yu Cheng, Francesco Cucca, Eco J. C. de Geus, Panos Deloukas, Michele K. Evans, Myriam Fornage, Yechiel Friedlander, Philippe Froguel, Leif Groop, Myron D. Gross, Tamara B. Harris, Caroline Hayward, Chew-Kiat Heng, Erik Ingelsson, Norihiro Kato, Bong-Jo Kim, Woon-Puay Koh, Jaspal S. Kooner, Antje Körner, Diana Kuh, Johanna Kuusisto, Markku Laakso, Xu Lin, Yongmei Liu, Ruth J. F. Loos, Patrik K. E. Magnusson, Winfried März, Mark I. McCarthy, Albertine J. Oldehinkel, Ken K. Ong, Nancy L. Pedersen, Mark A. Pereira, Annette Peters, Paul M. Ridker, Charumathi Sabanayagam, Michele Sale, Danish Saleheen, Juha Saltevo, Peter EH. Schwarz, Wayne H. H. Sheu, Harold Snieder, Timothy D. Spector, Yasuharu Tabara, Jaakko Tuomilehto, Rob M. van Dam, James G. Wilson, James F. Wilson, Bruce H. R. Wolffenbuttel, Tien Yin Wong, Jer-Yuarn Wu, Jian-Min Yuan, Alan B. Zonderman, Nicole Soranzo, Xiuqing Guo, David J. Roberts, Jose C. Florez, Robert Sladek, Josée Dupuis, Andrew P. Morris, E-Shyong Tai, Elizabeth Selvin, Jerome I. Rotter, Claudia Langenberg, Inês Barroso, James B. Meigs

**Affiliations:** 1 Department of Human Genetics, Wellcome Trust Sanger Institute, Genome Campus, Hinxton, Cambridge, United Kingdom; 2 Division of General Internal Medicine, Massachusetts General Hospital, Boston, MA, United States of America; 3 Harvard Medical School, Boston, MA, United States of America; 4 Department of Biostatistics, Boston University School of Public Health, Boston, MA, United States of America; 5 Department of Population Medicine, Harvard Medical School, Harvard Pilgrim Health Care Institute, Boston, MA, United States of America; 6 Massachusetts General Hospital, Boston, MA, United States of America; 7 Cardiovascular Medicine Unit, Department of Medicine Solna, Karolinska Institutet, Stockholm, Sweden; 8 Centre for Molecular Medicine, Karolinska Universitetsjukhuset, Solna, Sweden; 9 MRC Epidemiology Unit, Institute of Metabolic Science, University of Cambridge School of Clinical Medicine, Cambridge, United Kingdom; 10 Department of Internal Medicine, Lausanne University Hospital (CHUV), Lausanne, Switzerland; 11 Department of Epidemiology, The Johns Hopkins Bloomberg School of Public Health, Baltimore, MD, United States of America; 12 Division of Nephrology, University of Utah, Salt Lake City, UT, United States of America; 13 Department of Human Genetics, University of Utah, Salt Lake City, UT, United States of America; 14 Institute for Translational Genomics and Population Sciences, Department of Pediatrics, LABioMed at Harbor-UCLA Medical Center, Torrance, CA, United States of America; 15 Saw Swee Hock School of Public Health, National University of Singapore, Singapore, Singapore; 16 National Heart, Lung, and Blood Institute's Framingham Heart Study, Framingham, MA, United States of America; 17 Division of Preventive Medicine, Brigham and Women's Hospital and Harvard Medical School, Boston, MA, United States of America; 18 Department of Epidemiology and Biostatistics, School of Public Health, Imperial College London, London, United Kingdom; 19 Department of Cardiology, Ealing Hospital NHS Trust, Southall, Middlesex, United Kingdom; 20 Life Sciences Institute, National University of Singapore, Singapore, Singapore; 21 Phoenix Epidemiology and Clinical Research Branch, National Institute of Diabetes and Digestive and Kidney Diseases, National Institutes of Health, Phoenix, AZ, United States of America; 22 Key Laboratory of Pathobiology, Ministry of Education, Jilin University, Changchun, Jilin, China; 23 College of Basic Medical Sciences, Jilin University, Changchun, Jilin, China; 24 Division of General Internal Medicine, The Johns Hopkins University School of Medicine, Baltimore, MD, United States of America; 25 Welch Center for Prevention, Epidemiology and Clinical Research, The Johns Hopkins University, Baltimore, MD, United States of America; 26 Division of Epidemiology, Department of Medicine, Vanderbilt University Medical Center, Nashville, TN, United States of America; 27 Institute of Biomedical Sciences, Academia Sinica, Taipei, Taiwan; 28 University of Virginia Center for Public Health Genomics, Charlottesville, VA, United States of America; 29 Personalised Healthcare & Biomarkers, Innovative Medicines and Early Development Biotech Unit, AstraZeneca, Cambridge, United Kingdom; 30 California Pacific Medical Center Research Institute, San Francisco, CA, United States of America; 31 Centre for Quantitative Medicine, Duke-NUS Medical School, Singapore, Singapore; 32 Division of Structural and Functional Genomics, Center for Genome Science, Korean National Institute of Health, Osong, Chungchungbuk-do, South Korea; 33 Biological Psychology, Vrije Universiteit Amsterdam, Amsterdam, Netherlands; 34 The Key Laboratory of Nutrition and Metabolism, Institute for Nutritional Sciences, Shanghai Institutes for Biological Sciences, Chinese Academy of Sciences, University of the Chinese Academy of Sciences, Shanghai, People’s Republic of China; 35 Department of Biostatistics and Center for Statistical Genetics, University of Michigan, Ann Arbor, MI, United States of America; 36 William Harvey Research Institute, Barts and The London School of Medicine and Dentistry, Queen Mary University of London, London, United Kingdom; 37 Vth Department of Medicine, Medical Faculty Mannheim, University of Heidelberg, Mannheim, Germany; 38 Department of Immunology, Genetics and Pathology, Science for Life Laboratory, Uppsala University, Uppsala, Sweden; 39 Lund University Diabetes Centre, Lund University, Lund, Sweden; 40 University of Lille, CNRS, Institut Pasteur of Lille, UMR 8199—EGID, Lille, France; 41 Singapore Eye Research Institute, The Academia Level 6, Discovery Tower, Singapore, Singapore; 42 The Charles Bronfman Institute for Personalized Medicine, The Icahn School of Medicine at Mount Sinai, New York, NY, United States of America; 43 The Genetics of Obesity and Related Metabolic Traits Program, The Icahn School of Medicine at Mount Sinai, New York, NY, United States of America; 44 Wellcome Trust Centre for Human Genetics, University of Oxford, Oxford, United Kingdom; 45 Institute of Epidemiology II, Research Unit of Molecular Epidemiology, Helmholtz Zentrum München, German Research Center for Environmental Health, Neuherberg, Germany; 46 German Center for Diabetes Research (DZD e.V.), Partner Munich, Munich, Germany; 47 Data Tecnica International, Glen Echo, MD, United States of America; 48 Laboratory of Neurogenetics, National Institute on Aging, Bethesda, MD, United States of America; 49 MRC Human Genetics Unit, Institute of Genetics and Molecular Medicine, University of Edinburgh, Western General Hospital, Edinburgh, Scotland; 50 Department of Epidemiology, University of Groningen, University Medical Center Groningen, Groningen, Netherlands; 51 Data Coordinating Center, Boston University School of Public Health, Boston, MA, United States of America; 52 Istituto di Ricerca Genetica e Biomedica (IRGB), CNR, Monserrato, Italy; 53 Department of Preventive Medicine, University of Southern California, Los Angeles, CA, United States of America; 54 Department of Gene Diagnostics and Therapeutics, Research Institute, National Center for Global Health and Medicine, Tokyo, Japan; 55 Department of Endocrinology, University of Groningen, University Medical Center Groningen, Groningen, Netherlands; 56 MRC Unit for Lifelong Health & Ageing, London, United Kingdom; 57 Division of Cardiovascular Medicine, Radcliffe Department of Medicine, University of Oxford, Oxford, United Kingdom; 58 Spanish Biomedical Research Centre in Diabetes and Associated Metabolic Disorders (CIBERDEM), Instituto de Investigación Sanitaria del Hospital Clínico San Carlos (IdISSC), Madrid, Spain; 59 Institute for Community Medicine, University Medicine Greifswald, Greifswald, Germany; 60 National Institute for Health and Welfare (THL), Helsinki, Finland; 61 University of Helsinki, Institute for Molecular Medicine, Finland (FIMM) and Diabetes and Obesity Research Program, Helsinki, Finland; 62 Translational Gerontology Branch, National Institute on Aging, Baltimore, MD, United States of America; 63 Department of Clinical Epidemiology, Biostatistics and Bioinformatics, Academic Medical Center, University of Amsterdam, Amsterdam, Netherlands; 64 Durrer Center for Cardiogenetic Research, ICIN-Netherlands Heart Institute, Utrecht, Netherlands; 65 Department of Epidemiology and Prevention, Wake Forest School of Medicine, Winston-Salem, NC, United States of America; 66 Integrated Research and Treatment (IFB) Center Adiposity Diseases, University of Leipzig, Leipzig, Germany; 67 Centre for Global Health Research, Usher Institute of Population Health Sciences and Informatics, University of Edinburgh, Edinburgh, Scotland; 68 Laboratory of Clinical Investigation, National Institute on Aging, Baltimore, MD, United States of America; 69 School of Chinese Medicine, China Medical University, Taichung City, Taiwan; 70 Department of Biomedical Science, Hallym University, Chuncheon, Gangwon-do, South Korea; 71 Department of Nutrition Sciences, University of Alabama at Birmingham and the Birmingham Veterans Affairs Medical Center, Birmingham, AL, United States of America; 72 Division of Endocrinology, Diabetes, and Metabolism, Department of Medicine, Cedars-Sinai Medical Center, Los Angeles, CA, United States of America; 73 Department of Psychiatry, University of Groningen, University Medical Center Groningen, Groningen, Netherlands; 74 Institute for Clinical Diabetology, German Diabetes Center, Leibniz Institute for Diabetes Research at Heinrich Heine University Düsseldorf, Düsseldorf, Germany; 75 German Center for Diabetes Research (DZD), München-Neuherberg, Germany; 76 Division of Endocrinology, Diabetes, Metabolism, Department of Internal Medicine, Wexner Medical Center, The Ohio State University, Columbus, OH, United States of America; 77 Boston VA Research Institute, Inc., Boston, MA, United States of America; 78 Department of Geriatric Medicine, Ehime University Graduate School of Medicine, Ehime, Japan; 79 Department of Clinical Gene Therapy, Osaka University Graduate School of Medicine, Suita, Japan; 80 Department of Geriatric Medicine and Nephrology, Osaka University Graduate School of Medicine, Suita, Japan; 81 Genome Institute of Singapore, Agency for Science Technology and Research, Singapore, Singapore; 82 Center of Pediatric Research, University Hospital for Children & Adolescents, Dept. of Women's & Child Health, University of Leipzig, Leipzig, Germany; 83 LIFE Child, LIFE Leipzig Research Center for Civilization Diseases, University of Leipzig, Leipzig, Germany; 84 Faculty of Collaborative Regional Innovation, Ehime University, Ehime, Japan; 85 Department of Medical Research, Taichung Veterans General Hospital, Taichung, Taiwan; 86 Institute of Human Genetics, Technische Universität München, Munich, Germany; 87 Institute of Human Genetics, Helmholtz Zentrum München, Neuherberg, Germany; 88 Munich Cluster for Systems Neurology (SyNergy), Munich, Germany; 89 Department of Epidemiology, Graduate School of Public Health, University of Pittsburgh, Pittsburgh, PA, United States of America; 90 Center for Evidence-based Healthcare, University Hospital and Medical Faculty Carl Gustav Carus, Dresden, Germany; 91 Institute of Genetic Epidemiology, Helmholtz Zentrum München—German Research Center for Environmental Health, Neuherberg, Germany; 92 Department of Medicine I, University Hospital Grosshadern, Ludwig-Maximilians-Universität, Munich, Germany; 93 DZHK (German Centre for Cardiovascular Research), partner site Munich Heart Alliance, Munich, Germany; 94 Laboratory of Genetics, National Institute on Aging, Baltimore, MD, United States of America; 95 Institute for Clinical Chemistry and Laboratory Medicine, University Medicine Greifswald, Greifswald, Germany; 96 Division of Epidemiology and Community Health, University of Minnesota, Minneapolis, MN, United States of America; 97 University of Split, Split, Croatia; 98 Centre for Population Health Sciences, University of Edinburgh, Edinburgh, United Kingdom; 99 Oxford Centre for Diabetes, Endocrinology and Metabolism, Radcliffe Department of Medicine, University of Oxford, Oxford, United Kingdom; 100 Department of Genomics of Common Disease, School of Public Health, Imperial College London, London, United Kingdom; 101 Department of Medicine, Medical University of South Carolina, Charleston, SC, United States of America; 102 Department of Preventive Medicine, Northwestern University Feinberg School of Medicine, Chicago, IL, United States of America; 103 Center for Public Health Genomics, University of Virginia School of Medicine, Charlottesville, VA, United States of America; 104 Wellcome Trust Centre for Human Genetics, Nuffield Department of Medicine, University of Oxford, Oxford, United Kingdom; 105 Department of Endocrinology and Diabetology, University Hospital Düsseldorf, Düsseldorf, Germany; 106 INSERM, UMR_S 1138, Centre de Recherche des Cordelier, Paris, France; 107 Université Paris Diderot, Sorbonne Paris Cité, UFR de Médecine, Paris, France; 108 Assistance Publique Hôpitaux de Paris, Bichat Hospital, DHU FIRE, Department of Diabetology, Endocrinology and Nutrition, Paris, France; 109 University of Edinburgh, Edinburgh, United Kingdom; 110 Science for life laboratory, Karolinska Institutet, Solna, Sweden; 111 The New York Academy of Medicine, New York, NY, United States of America; 112 Institute of Medical Informatics, Biometry and Epidemiology, Ludwig-Maximilians-Universität, Munich, Germany; 113 Department of Genetics, University of Groningen, University Medical Center Groningen, Groningen, Netherlands; 114 Health Disparities Unit, National Institute on Aging, National Institutes of Health, Baltimore, MD, United States of America; 115 Department of Statistics and Applied Probability, National University of Singapore, Singapore, Singapore; 116 NUS Graduate School for Integrative Science and Engineering, National University of Singapore, Singapore, Singapore; 117 Department of Medicine; University of Leipzig, Leipzig, Germany; 118 Dept of Community Medicine, Faculty of Health Sciences, University of Tromsø, Tromsø, Norway; 119 Institute of Cardiovascular Science, University College London, London, United Kingdom; 120 Department of Vascular Medicine, Academic Medical Center, Amsterdam, Netherlands; 121 Department of Epidemiology and Public Health, University College London, London, United Kingdom; 122 Institute for Social and Economic Research, University of Essex, Colchester, United Kingdom; 123 Pat Macpherson Centre for Pharmacogenetics and Pharmacogenomics, Medical Research Institute, Ninewells Hospital and Medical School, Dundee, United Kingdom; 124 Ophthalmology and Visual Sciences Academic Clinical Program (Eye ACP), Duke-NUS Medical School, Singapore, Singapore; 125 Department of Ophthalmology, Yong Loo Lin School of Medicine, National University of Singapore, Singapore, Singapore; 126 Singapore National Eye Centre, Singapore, Singapore; 127 Dept of Medicine III, University of Dresden, Medical Faculty Carl Gustav Carus, Dresden, Germany; 128 Imperial College Healthcare NHS Trust, London, United Kingdom; 129 Division of Genetics, Brigham and Women's Hospital and Harvard Medical School, Boston, MA, United States of America; 130 Broad Institute of MIT and Harvard, Cambridge, MA, United States of America; 131 Dipartimento di Scienze Biomediche, Università di Sassari, Italy; 132 Princess Al-Jawhara Al-Brahim Centre of Excellence in Research of Hereditary Disorders (PACER-HD), King Abdulaziz University, Jeddah, Saudi Arabia; 133 Brown Foundation Institute of Molecular Medicine, Division of Epidemiology, School of Public Health, University of Texas Health Science Center at Houston, Houston, TX, United States of America; 134 Braun School of Public Health, Hebrew University-Hadassah Medical Center, Jerusalem, Israel; 135 CNRS 8199-Lille University, France; 136 Finnish Institute for Molecular Medicine (FIMM), Helsinki, Finland; 137 Department of Laboratory Medicine and Pathology, University of Minnesota, Minneapolis, MN, United States of America; 138 National Institute on Aging, Bethesda, MD, United States of America; 139 Institute of Genetics and Molecular Medicine, University of Edinburgh, Edinburgh, United Kingdom; 140 Department of Paediatrics, Yong Loo Lin School of Medicine, National University of Singapore, Singapore, Singapore; 141 Khoo Teck Puat-National University Children’s Medical Institute, National University Health System, Singapore, Singapore; 142 Department of Medical Sciences, Molecular Epidemiology and Science for Life Laboratory, Uppsala University, Uppsala, Sweden; 143 Department of Medicine, Division of Cardiovascular Medicine, Stanford University School of Medicine, Stanford, CA, United States of America; 144 Duke-NUS Medical School Singapore, Singapore; 145 National Heart and Lung Institute, Imperial College London, Hammersmith Hospital Campus, London, United Kingdom; 146 Institute of Clinical Medicine, Internal Medicine, University of Eastern Finland and Kuopio University Hospital, Kuopio, Finland; 147 Department of Epidemiology and Prevention, Division of Public Health Sciences, Wake Forest University, Winston-Salem, NC, United States of America; 148 The Mindich Child Health Development Institute, The Icahn School of Medicine at Mount Sinai, New York, NY, United States of America; 149 Department of Medical Epidemiology and Biostatistics, Karolinska Insitutet, Stockholm, Sweden; 150 Clinical Institute of Medical and Chemical Laboratory Diagnostics, Medical University of Graz, Graz, Austria; 151 Synlab Academy, Synlab Services GmbH, Mannheim, Germany; 152 Oxford NIHR Biomedical Research Centre, Oxford University Hospitals Trust, Oxford, United Kingdom; 153 Division of Cardiovascular Medicine, Brigham and Women's Hospital and Harvard Medical School, Boston, MA, United States of America; 154 Department of Biostatistics and Epidemiology, University of Pennsylvania, Philadelphia, PA, United States of America; 155 Center for Non-Communicable Diseases, Karachi, Pakistan; 156 Department of Medicine, Central Hospital, Central Finland, Jyväskylä, Finland; 157 Division of Endocrine and Metabolism, Department of Internal Medicine, Taichung Veterans General Hospital, Taichung, Taiwan; 158 School of Medicine, National Yang-Ming University, Taipei, Taiwan; 159 School of Medicine, National Defense Medical Center, Taipei, Taiwan; 160 Department of Twin Research and Genetic Epidemiology, King’s College London, London, United Kingdom; 161 Center for Genomic Medicine, Kyoto University Graduate School of Medicine, Kyoto, Japan; 162 Chronic Disease Prevention Unit, National Institute for Health and Welfare, Helsinki, Finland; 163 Dasman Diabetes Institute, Dasman, Kuwait; 164 Centre for Vascular Prevention, Danube-University Krems, Krems, Austria; 165 Saudi Diabetes Research Group, King Abdulaziz University, Fahd Medical Research Center, Jeddah, Saudi Arabia; 166 Department of Physiology and Biophysics, University of Mississippi Medical Center, Jackson, MS, United States of America; 167 Division of Cancer Control and Population Sciences,University of Pittsburgh Cancer Institute, Pittsburgh, PA, United States of America; 168 Laboratory of Epidemiology & Population Sciences, National Institute on Aging, National Institutes of Health, Baltimore, MD, United States of America; 169 Department of Haematology, University of Cambridge, Cambridge, United Kingdom; 170 The National Institute for Health Research Blood and Transplant Unit (NIHR BTRU) in Donor Health and Genomics at the University of Cambridge, United Kingdom; 171 Biomedical Research Centre Oxford Haematology Theme and Radcliffe Department of Medicine, University of Oxford, John Radcliffe Hospital, Headley Way, Headington, Oxford, United Kingdom; 172 NHS Blood and Transplant, Headington, Oxford, United Kingdom; 173 Diabetes Unit and Center for Human Genetic Research, Massachusetts General Hospital, Boston, MA, United States of America; 174 Programs in Metabolism and Medical & Population Genetics, Broad Institute, Cambridge, MA, United States of America; 175 Department of Medicine, McGill University, Montreal, Quebec, Canada; 176 Department of Biostatistics, University of Liverpool, Liverpool, United Kingdom; 177 Department of Medicine, Yong Loo Lin School of Medicine, National University of Singapore, Singapore; 178 Institute of Metabolic Science, University of Cambridge, Cambridge, United Kingdom; Centers for Disease Control and Prevention, UNITED STATES

## Abstract

**Background:**

Glycated hemoglobin (HbA1c) is used to diagnose type 2 diabetes (T2D) and assess glycemic control in patients with diabetes. Previous genome-wide association studies (GWAS) have identified 18 HbA1c-associated genetic variants. These variants proved to be classifiable by their likely biological action as erythrocytic (also associated with erythrocyte traits) or glycemic (associated with other glucose-related traits). In this study, we tested the hypotheses that, in a very large scale GWAS, we would identify more genetic variants associated with HbA1c and that HbA1c variants implicated in erythrocytic biology would affect the diagnostic accuracy of HbA1c. We therefore expanded the number of HbA1c-associated loci and tested the effect of genetic risk-scores comprised of erythrocytic or glycemic variants on incident diabetes prediction and on prevalent diabetes screening performance. Throughout this multiancestry study, we kept a focus on interancestry differences in HbA1c genetics performance that might influence race-ancestry differences in health outcomes.

**Methods & findings:**

Using genome-wide association meta-analyses in up to 159,940 individuals from 82 cohorts of European, African, East Asian, and South Asian ancestry, we identified 60 common genetic variants associated with HbA1c. We classified variants as implicated in glycemic, erythrocytic, or unclassified biology and tested whether additive genetic scores of erythrocytic variants (GS-E) or glycemic variants (GS-G) were associated with higher T2D incidence in multiethnic longitudinal cohorts (*N* = 33,241). Nineteen glycemic and 22 erythrocytic variants were associated with HbA1c at genome-wide significance. GS-G was associated with higher T2D risk (incidence OR = 1.05, 95% CI 1.04–1.06, per HbA1c-raising allele, *p* = 3 × 10^−29^); whereas GS-E was not (OR = 1.00, 95% CI 0.99–1.01, *p* = 0.60). In Europeans and Asians, erythrocytic variants in aggregate had only modest effects on the diagnostic accuracy of HbA1c. Yet, in African Americans, the X-linked *G6PD* G202A variant (T-allele frequency 11%) was associated with an absolute decrease in HbA1c of 0.81%-units (95% CI 0.66–0.96) per allele in hemizygous men, and 0.68%-units (95% CI 0.38–0.97) in homozygous women. The *G6PD* variant may cause approximately 2% (*N* = 0.65 million, 95% CI 0.55–0.74) of African American adults with T2D to remain undiagnosed when screened with HbA1c. Limitations include the smaller sample sizes for non-European ancestries and the inability to classify approximately one-third of the variants. Further studies in large multiethnic cohorts with HbA1c, glycemic, and erythrocytic traits are required to better determine the biological action of the unclassified variants.

**Conclusions:**

As G6PD deficiency can be clinically silent until illness strikes, we recommend investigation of the possible benefits of screening for the *G6PD* genotype along with using HbA1c to diagnose T2D in populations of African ancestry or groups where *G6PD* deficiency is common. Screening with direct glucose measurements, or genetically-informed HbA1c diagnostic thresholds in people with G6PD deficiency, may be required to avoid missed or delayed diagnoses.

## Abstract 2

Ines Barroso and colleagues identify a genetic variant that leads to reduced levels of HbA1c in African American adults; 2% of this population are at risk of missed diagnosis for diabetes.

## Author summary

**Why was this study done?:**

- Blood glucose binds in an irreversible manner to circulating hemoglobin in red blood cells (RBCs), generating “glycated hemoglobin,” called HbA1c. HbA1c is used to diagnose and monitor diabetes.
- Previous large-scale human genetic studies have demonstrated that HbA1c is influenced by genetic variants. Some variants are thought to influence the function, structure, and lifespan of the red blood itself (“erythrocytic variants”), while others are thought to influence blood glucose control (“glycemic variants”). This study aimed to identify additional variants influencing HbA1c levels, and investigate the extent to which variants affecting this measurement independently of blood glucose concentration may lead to misdiagnosis, mistreatment, and human health disparities.

**What did the researchers do and find?:**

- We studied genetic variants and their association with HbA1c levels in almost 160,000 people from European, African, East Asian, and South Asian ancestry from 82 separate studies worldwide. We found 60 genetic variants influencing HbA1c, of which 42 variants were new. Of the 60 variants, we found 19 glycemic variants and 22 erythrocytic variants.
- In approximately 33,000 people from 5 ancestry groups followed carefully over time, we found that the more glycemic variants a person had, the higher their risk to get diabetes (OR = 1.05 per HbA1c-raising allele, *p* = 3 × 10^−29^). However, more erythrocytic variants did not lead to a higher risk of diabetes, meaning erythrocytic variants that lower HbA1c levels independently from glucose concentration could lead to missed diagnosis of diabetes.
- Next, we found that in everyone but those of African ancestry, those with more versus those with less of the 60 HbA1c genetic variants had a fairly small difference in HbA1c (about 0.2 units), while those of African ancestry had a larger difference (about 0.8 units, a fairly large number for this medical test).
- This difference in African ancestry was explained by one erythrocytic variant on the X chromosome. This variant mutates the protein made by the gene “glucose-6-phosphate dehydrogenase” (*G6PD*), which can shorten RBC lifespan, and thus lower HbA1c levels, no matter the blood glucose level.
- About 11% of people of African American ancestry carry at least one copy of this *G6PD* variant, while almost no one of any other ancestry does. We estimated that if we tested all Americans for diabetes using HbA1c, about 650,000 African Americans would be missed because of these genetically lowered HbA1c levels.

**What do these findings mean?:**

- We may want to investigate the benefits of screening for the *G6PD* genotype in specific communities or perform additional diagnostic tests to avoid health disparities between communities.
- It will also be important to follow up with additional studies to check whether new standardized thresholds for diagnoses should be recommended for those that have this *G6PD* variant.

## Introduction

Type 2 diabetes (T2D) is a health scourge rising unabated worldwide, escaping all past and current control measures, in part because only half of prevalent T2D worldwide has been clinically diagnosed [1]. Glycated hemoglobin (HbA1c) is an accepted diagnostic test for T2D and a principal clinical measure of glycemic control in individuals with diabetes. T2D arises from the environment interacting with genetics. Studies investigating genetic contributions to HbA1c in individuals of European [2–4] and Asian ancestry [5–7] have identified 18 loci influencing HbA1c through glycemic and nonglycemic pathways, the latter primarily reflecting erythrocytic biology. Alterations in HbA1c that are due to genetic variation acting through nonglycemic pathways may not accurately reflect ambient glycemia or T2D risk and could affect the validity of HbA1c as a diagnostic test and measure of glycemic control in some individuals or populations. Some genetic variants (e.g., the sickle cell variant HbS) that vary in frequency across ancestries can interfere with the accuracy of certain assays [8]. Further, certain hematologic conditions associated with shortened erythrocyte lifespan (e.g., hemolytic anemias) lower HbA1c values irrespective of the assay performed. HbA1c values in such patients may no longer accurately reflect ambient glycemia [9].

Epidemiologic studies have reported ethnic differences in HbA1c, with African Americans having, on average, higher HbA1c than European ancestry Americans [10]. While these differences are largely due to demographic and metabolic factors [11,12], genetic factors associated with hematologic conditions that impact erythrocyte turnover may confound the relationship between HbA1c and glycemia, causing misclassification of T2D diagnosis [8,13].

This study had 3 aims, the first was to expand genetic discovery efforts to larger sample sizes, including populations of ancestries not previously studied, to uncover novel loci influencing HbA1c and that might capture a greater fraction of the variability in HbA1c. Second, as done in previous studies, we aimed to classify the variants as acting through glycemic or erythrocytic biology. Thirdly, as erythrocytic variants may influence HbA1c due to effects on the red blood cell (RBC), we wished to explore whether this might lead to HbA1c values that no longer reflected ambient glycemia. To do this, we specifically tested the hypothesis that HbA1c-associated genetic variants, particularly those that act through erythrocytic pathways, influence the performance of HbA1c for diabetes risk prediction and diabetes diagnoses (S1 Fig).

## Methods

Analysis plans were followed and can be found in S1 Analysis Plans.

### Genetic discovery study participants

In the genetic discovery analysis, we combined data from up to 159,940 participants (maximum number available for any variant) of European, African American, East Asian, and South Asian ancestry, including subsets from previous publications [4,5] (S1 Table, S2 Fig). All participants were free of diabetes defined by physician diagnosis, medication use, or fasting glucose (FG) ≥ 7 mmol/L. A small number of cohorts also removed individuals with 2hr glucose (2hrGlu) ≥ 11.1 mmol/L, or HbA1c ≥ 6.5%, where FG was not available (details of exclusions by individual cohorts, S1 Table). Analysis followed the details in S1 Analysis Plans (Hemoglobin A1c Genetic Discovery Analysis Plan).

### HbA1c measurement

Where possible, studies reported HbA1c as a National Glycohemoglobin Standardization Program (NGSP) percent [14] (S1 Table).

### Genotyping and quality control

Each cohort was genotyped on commercially available genome-wide arrays (for instance, the Affymetrix Genome-Wide Human SNP Assay 6.0 or the Illumina Human610-Quad BeadChip) or the Illumina CardioMetabochip (Metabochip) [15]. Variant and sample quality control (QC) was conducted within each cohort following a shared analysis plan (S1 Analysis Plans). Cohorts were advised to keep SNPs with hardy-weinberg-disequilibrium *p*-value ≥ 1 × 10^−6^, SNP genotyping call rate ≥ 95% and minor allele frequency (MAF) ≥ 1% (full details of SNP and sample QC can be found in S1 Table). Following QC, studies with genome-wide array data were imputed (primarily using the Phase 2 of the International HapMap Project reference panel [16], see S1 Table, row 40), and poorly imputed variants (variants which could not reliably be inferred from surrounding variants) were excluded based on standard imputation quality thresholds (R-sq < 0.3, INFO < 0.4). Approximately 2.5 million SNPs were available for analysis after imputation and QC (S1 Table). QC of the Metabochip data is described elsewhere, but included filtering out poorly genotyped individuals or low-quality SNPs [17]. Variant association testing in men and women combined was conducted under an additive model adjusting for study-specific covariates and was limited to variants with MAF of at least 1% in each cohort. Details of the study cohorts, genotyping platforms and QC criteria, imputation reference panel, covariates in the analysis, and software used are provided for each study in S1 Table. Our study followed STREGA guidelines (S1 Checklist).

### Genetic discovery using ancestry-specific and trans-ancestry meta-analyses

Association data were combined within each ancestry group using a fixed-effects meta-analysis in METAL, which assumes the SNP effect is the same for each study within an ancestry [18]. Results for each cohort were corrected for any systematic biases, such as residual population structure using the genomic control inflation factor, λ_GC_ [17,19]. We excluded variants from further follow-up if they had an ancestry-specific sample size *N* < 20,000 in Europeans, *N* < 3,000 in African Americans, *N* < 7,000 in East Asians, and *N* < 3,000 in South Asians (minimum number of samples, where the threshold was chosen to minimize signals driven by a single cohort), or evidence of significant within-ancestry heterogeneity, suggesting effect size significantly differs between cohorts of the same ancestry (Cochran’s Q-test heterogeneity *p*-value < 0.0001). We retained the lead variant in the X-chromosome analysis of the African American ancestry data (rs1050828, G202A in *G6PD*) despite significant heterogeneity, as it was a strong biological candidate.

Ancestry-specific meta-analysis results were conservatively corrected for a second round of genomic control by ancestry: European (λ_GC_ = 1.072); African American (λ_GC_ = 1.020); East Asian (λ_GC_ = 1.027); South Asian (λ_GC_ = 1.004); and combined using the Meta-Analysis of Transethnic Association (MANTRA) software that accounts for allelic heterogeneity across ancestry groups [20].

### Identification of primary and secondary distinct HbA1c-associated signals

Variants were considered to be significantly associated with HbA1c when they met standard genome-wide significant thresholds (based on *p* = 0.05 divided by the estimated number of independent tests across the genome), of *p* < 5 × 10^−8^ in the European and Asian, or *p* < 2.5 × 10^−8^ in African American [21] ancestry-specific meta-analyses, or a log_10_ Bayes Factor ≥6 in the transancestry meta-analysis. All significant variants within 500 kb of a lead (most significantly associated) variant were grouped into a single locus. Novel loci were by definition >500 kb from previously reported HbA1c-associated variants. We ran approximate conditional analyses using the Genome-wide Complex Trait Analysis (GCTA) software [22,23] (following analysis plans detailed in S1 Analysis Plans, Conditional analyses in GCTA) using the Women’s Genome Health Study (WGHS, Europeans), Jackson Heart Study (JHS, African Americans), Singapore Malay Eye Study (SiMES, East Asians), and the London Life Sciences Prospective Population Study (LOLIPOP, South Asians) as reference populations for linkage disequilibrium (LD) estimates, to confirm the lead variants on the autosomes (within 1 Mb) were distinct, and similarly used exact conditional regression for the African-American signals on the X-chromosome in JHS.

To identify distinct signals at associated loci (that is, secondary signals), we performed approximate conditional analyses using GCTA, conditioning on lead variants identified in the transancestry MANTRA analysis. Where the lead variant was absent in a cohort, an exact proxy (r^2^ = 1) was used, unless the variant was very low frequency or monomorphic.

### Classification of variants as glycemic or erythrocytic

We extracted summary association statistics from publicly available meta-analysis results for glycemic [17,24–26] and blood-cell [27] traits and asked a subset of the genome-wide discovery cohorts to repeat association analyses for each lead variant, conditioning on any one of FG, 2hrGlu, hemoglobin level (Hb), mean corpuscular volume (MCV), or mean corpuscular hemoglobin (MCH), where available (S3 Fig, S2 Table and S3 Table).

Variants were classified as “glycemic” if they were associated (*p* < 0.0001) with any of the glycemic traits from publicly available results or had ≥25% attenuation of variant HbA1c effect size in association models conditioned on fasting or 2hrGlu. That is, evidence of being associated with any of the glycemic traits or a reduction in the effect of the variant on HbA1c after repeated association analysis in a model additionally adjusting for fasting/2hrGlu, suggested the observed association with HbA1c was being driven through an association with fasting/2hrGlu. Variants not classified as glycemic were classified as “erythrocytic” if they were associated (*p* < 0.0001) with Hb, MCH, MCV, PCV, RBC, or MCHC in the publicly available results or, as above, had ≥25% attenuation of effect size in Hb-, MCV-, or MCH-conditioned models (suggesting the observed association with HbA1c was being driven through an association with these blood cell traits). The 25% attenuation threshold was chosen as the optimal balance between specificity and sensitivity based on comparisons with the classification based only on association with any of the glycemic/erythrocytic traits. Two SNPs were classified based on evidence from the literature, rs12132919 (*TMEM79*) was classified as erythrocytic based on association with MCHC in Japanese individuals [28] and rs7616006 (*SYN2*) was classified as erythrocytic based on association with platelet count in Europeans [29].

Variants associated with HbA1c but not glycemic or erythrocytic traits remained “unclassified” (S3 Fig). A single variant (rs579459 near *ABO*) was classified as both glycemic and erythrocytic, but as we were primarily concerned about variants that might affect HbA1c without reflecting ambient glycemia and this variant also affected glycemia, we treated it as glycemic in all analyses.

### Effect of HbA1c genetic scores on reclassification of prevalent undiagnosed T2D for population screening using HbA1c

Analyses on the reclassification of prevalent T2D around the HbA1c 6.5% threshold before and after accounting for the contribution of erythrocytic variants were conducted in up to 19,380 individuals and incident T2D prediction analyses in up to 33,241 individuals from European, African, and East Asian ancestry cohorts (derived in part from discovery cohorts; in S4 Table, and following the details in the S1 Analysis Plans, Net-reclassification analysis). We acknowledge that nonindependent GWAS discovery and application cohorts can lead to inflated effect estimates [30]; however, this was not evident in our study, and effect estimates across all cohorts were similar with low heterogeneity.

We estimated reclassification of prevalent T2D status by HbA1c after accounting for the contribution of erythrocytic loci in 5 population-based cohorts with 3 ancestries partially overlapping with the discovery GWAS: the Framingham Heart Study (FHS), the Atherosclerosis Risk in Communities Study (ARIC), and the Multiethnic Study of Atherosclerosis (MESA) in individuals of European ancestry; ARIC and MESA in African Americans; and MESA, the Taiwan-Metabochip Study for Cardiovascular Disease (TAICHI), and the Singapore Prospective Study (SP2) in East Asians (*N* = 19,380). Variant-adjusted HbA1c was calculated as:

$$Y_{i}-\sum {\hat{\beta }}_{k}(g_{ki}-E(g_{ki}))$$

where *Y*_*i*_ was the measured HbA1c for individual, i, ${\hat{\beta }}_{i}$ is the ancestry-specific, meta-analytic *β* coefficient for the k^th^ erythrocytic SNP, *g*_*ki*_ is the dosage (estimated number of HbA1c-raising alleles), and E(*g*_*ki*_) was two times the HbA1c-raising allele frequency. When the less frequent (minor) allele was associated with higher HbA1c, it was coded as the HbA1c-raising allele, when it was associated with lower HbA1c, the more frequent (major) allele was coded as the HbA1c-raising allele. As some HbA1c-raising alleles in one ancestry could be HbA1c-lowering in a different ancestry, we coded HbA1c-raising alleles by ancestry.

Participants on antidiabetic therapy were excluded, and screen-detected T2D was defined as FG ≥ 7 mmol/L. For the reclassification analysis, we constructed 2-by-2 tables showing the proportion of participants reclassified around the HbA1c 6.5% diagnostic threshold, with and without adjusting measured HbA1c for the contribution of erythrocytic loci.

#### Calculation of genetic risk scores

Genetic risk scores of erythrocytic variants and glycemic variants (GS-E and GS-G, respectively) were calculated as detailed in S1 Analysis Plans (Investigate the Effect of Glycemic and Erythrocytic Hemoglobin A1c (HbA1c) Genetic Variants on Diabetes Prediction), as standard in the field, by summing the number of ancestry-specific HbA1c-raising alleles at each variant (0, 1, 2, or expected number of alleles based on the probability of each genotype), multiplied by their ancestry-specific β coefficients for HbA1c from the genome-wide association study (GWAS) meta-analysis multiplied by the number of variants and divided by the sum of β coefficients [31]. This means the contribution of each associated variant to the trait, in a given individual, is influenced by the number of “risk alleles” (or in this case HbA1c-raising alleles) and the effect of the variant on the trait (increase in HbA1c estimated from the meta-analysis).

### Effect of HbA1c genetic scores on prediction of incident T2D

We tested the hypothesis that glycemic and erythrocytic HbA1c loci predicted incident T2D differently in Europeans, East Asians, and African Americans from 5 cohorts (partially overlapping with the discovery GWAS) with prospective follow-up: FHS, the European Prospective Investigation into Cancer and Nutrition InterAct project (EPIC-InterAct), ARIC, MESA, and the Singapore Chinese Health Study (SCHS) (*N* = 33,241). Using age- and sex-adjusted regression models, we tested the association between the genetic scores GS-E or GS-G and incident T2D, defined by FG ≥ 7 mmol/L, 2hrGlu ≥ 11.1 mmol/L, antidiabetic medication use, or a physician diagnosis for T2D, accrued over a 10-to-15-year follow-up period. Clinical practice guidelines did not include HbA1c as a diagnostic test until 2010. As the majority of incident T2D cases were accrued before 2010, participants are very unlikely to have received a T2D diagnosis based only on HbA1c measurements. To test whether individuals with higher GS-E, compared to those with lower GS-E, had lower T2D risk for the same HbA1c, we adjusted models for baseline HbA1c. We meta-analyzed results using a fixed-effects meta-analysis and assessed heterogeneity using Higgin's I-squared. See S1 Analysis Plans (Investigate the Effect of Glycemic and Erythrocytic Hemoglobin A1c (HbA1c) Genetic Variants on Diabetes Prediction) for analysis plan.

### Ancestral differences in the genetic architecture of HbA1c

In FHS, ARIC, MESA, and SCHS, we calculated the difference in HbA1c of individuals at the bottom and top 5% of the distribution of an ancestry-specific GS composed of all 60 variants (GS-Total) and an equivalent analysis using GS-E.

We also pursued additional analyses at chromosome X rs1050828 because this single variant showed the largest effect on HbA1c in African Americans and was monomorphic in the other ancestries. The T allele is known to be associated with glucose-6-phosphate dehydrogenase (G6PD) deficiency, an enzymatic defect causing hemolytic anemia [32,33]. Imperfect correlation between HbA1c and glycemia may indicate the impact of reduced erythrocyte lifespan on HbA1c in individuals with the T allele. Fructosamine, a measure of serum protein glycation not influenced by erythrocyte-related factors, reflects average glycemia over the previous 2–3 weeks. Following the analysis plan detailed in S1 Analysis Plans (The Difference Between Fructosamine-inferred HbA1c and Measured HbA1c) we thus calculated the estimated residuals from a linear regression of HbA1c on fructosamine in ARIC African Americans (*N* = 1,676) to determine whether the T allele was associated with lower HbA1c than predicted by fructosamine, suggesting that the T allele artificially lowered HbA1c through a reduction in the average erythrocytic lifespan. We then reported the mean estimated residuals by genotype (women: CC, CT, TT; men: C, T).

#### Estimated number of African Americans with T2D in the United States whose diagnosis would be missed due to the *G6PD* variant if screened with HbA1c

Using publicly-available data from the National Health and Nutrition Examination Survey (NHANES) 2013–2014 [34], a nationally representative sample of US residents, we calculated the proportion of African American adults (aged ≥ 18 years) with T2D who would be missed by not accounting for rs1050828 when using a single HbA1c diagnostic threshold of 6.5%, assuming the observed effect size of rs1050828, allele frequency of 11% and accounting for NHANES sampling design. The study sample was restricted to 1,133 adults, aged ≥ 18 years, who self-identified as non-Hispanic black with measured HbA1c in 2013–2014. We defined known T2D by self-reported physician diagnosis or medication use. Assuming Hardy-Weinberg Equilibrium and a T allele frequency of 11% for the *G6PD* variant in our sample, we lowered the diagnostic threshold from the widely accepted 6.5%-units cut-point to 5.7%-units in men with the T genotype, 5.8%-units in women with the TT genotype, and 6.2%-units in women with the CT genotype. We then calculated the proportion of African American individuals with missed T2D diagnosis if screened with HbA1c using the 6.5% diagnostic threshold. We applied procedures to account for sampling probabilities and complex sampling design to enable population-level inferences. Data analysis was performed using SAS (version 9.2 or 9.3; SAS Institute, Cary, NC).

## Results

### HbA1c-associated genetic variants and classification into glycemic and nonglycemic pathways

**Fig 1:**
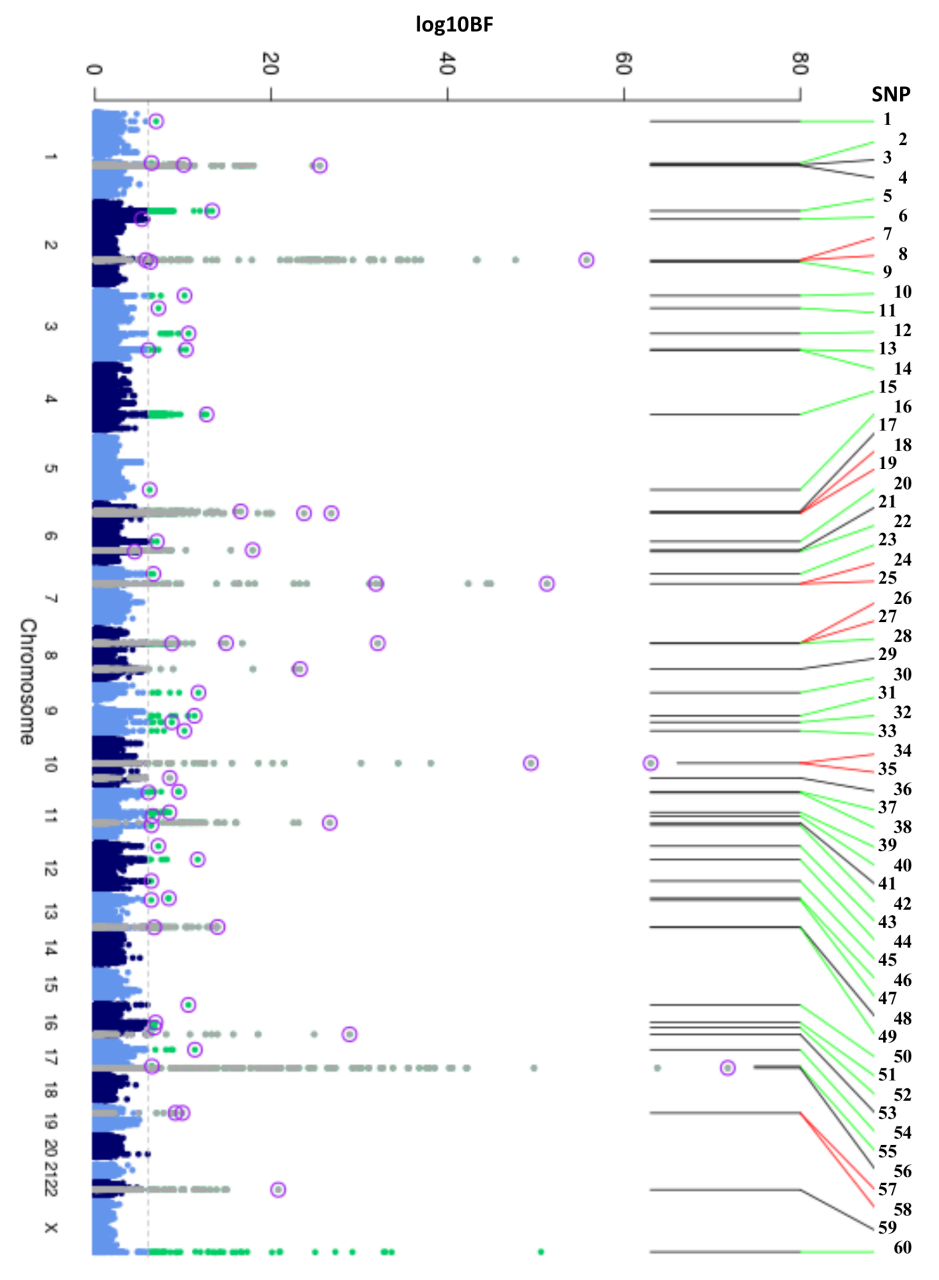
Manhattan plot of HbA1c associated variants. Manhattan plot of the transethnic meta-analysis results in MANTRA. The dashed grey line indicates log_10_BF = 6. Grey and green points denote known/novel loci, respectively. The lead HbA1c-associated variants identified through the ancestry-specific/transethnic analyses are circled in purple (the *G6PD* variant was not included in the MANTRA analysis, but the locus on the X-chromosome is indicated in the figure). Lines joining the plot & SNP number denote known loci (black), novel loci (green), and loci with a secondary distinct signal (red). MANTRA, Meta-Analysis of Transethnic Association.

To discover new genetic loci influencing HbA1c in populations from 4 different ancestries (European, African American, East Asian, and South Asian), we performed within-ancestry fixed-effects genome-wide association meta-analyses and transancestry meta-analyses using a model that allowed for different effects between ancestry groups (Methods, S2 Fig). Using this approach in up to 159,940 participants without diabetes, we identified 60 variants associated with HbA1c at genome-wide significance (Fig 1, Table 1 and S5 Table). Of 60, 18 have been previously reported, and 42 were novel, including distinct secondary signals at 5 known loci. To classify the associated loci into groups reflecting their likely mode of action on HbA1c, we repeated association analyses conditioning on erythrocytic or glycemic traits and performed lookups in publicly-available association results summary statistics for additional glycemic and erythrocytic traits (Methods, S3 Fig, S2 Table and S3 Table). Based on the combined results from conditional and lookup results, we were able to classify 22 variants as erythrocytic and 19 as glycemic, with 19 remaining unclassified (Fig 1, Table 1 and S5 Table).

**Table 1:** Table of HbA1c associated variants. Table with results and classification of the 60 HbA1c-associated variants. SNP number corresponds to number in Fig 1.

SNP	Markername	Chr.	Position (bp)	Effect Allele	Other Allele	Gene	Status	Signals	Classification	European ancestry METAL *p*-value	Trans-ethnic MANTRA log10BF
1	**rs2375278**	1	25401625	A	G	SYF2	Novel	Single	Unclassified	2.03 × 10^−7^	6.93
2	**rs267738**	1	149207249	T	G	CERS2	Novel	Single	Unclassified	2.59 × 10^−9^	6.41
3	**rs12132919**	1	154584765	A	C	TMEM79	Known	Single	**Erythrocytic**	0.0169	10.08
4	**rs857691**	1	156893002	T	C	SPTA1	Known	Single	**Erythrocytic**	3.97 × 10^−25^	25.52
5	**rs17509001**	2	23874735	C	T	ATAD2B	Novel	Single	Unclassified	1.94 × 10^−15^	13.30
6	**rs12621844**	2	48268239	T	C	FOXN2	Novel	Single	Unclassified	1.87 × 10^−8^	5.32
7	**rs13387347**	2	169463092	T	C	G6PC2	Novel	Multiple	**Glycemic**	0.308	5.77
8	**rs560887**	2	169471394	C	T	G6PC2	Known	Multiple	**Glycemic**	1.48 × 10^−58^	55.77
9	**rs17256082**	2	175000610	C	T	SCRN3	Novel	Single	Unclassified	0.00112	6.27
10	**rs7616006**	3	12242648	A	G	SYN2	Novel	Single	**Erythrocytic**	5.07 × 10^−10^	10.16
11	**rs9818758**	3	49357929	A	G	USP4	Novel	Single	Unclassified	7.74 × 10^−10^	7.20
12	**rs11708067**	3	124548468	A	G	ADCY5	Novel	Single	**Glycemic**	1.42 × 10^−12^	10.62
13	**rs8192675**	3	172207577	T	C	SLC2A2	Novel	Single	**Glycemic**	1.38 × 10^−11^	10.33
14	**rs4894799**	3	173278234	A	G	FNDC3B	Novel	Single	Unclassified	1.80 × 10^−6^	6.05
15	**rs13134327**	4	144879245	A	G	FREM3	Novel	Single	**Glycemic**	2.64 × 10^−15^	12.66
16	**rs11954649**	5	156988069	G	C	SOX30	Novel	Single	Unclassified	NA	6.20
17	**rs7756992**	6	20787688	G	A	CDKAL1	Known	Single	**Glycemic**	2.80 × 10^−12^	16.53
18	**rs1800562**	6	26201120	G	A	HFE	Known	Multiple	**Erythrocytic**	4.67 × 10^−28^	26.81
19	**rs198846**	6	26215442	G	A	HFE	Novel	Multiple	**Erythrocytic**	1.18 × 10^−23^	23.72
20	**rs11964178**	6	109668728	A	G	C6orf183	Novel	Single	**Erythrocytic**	6.38 × 10^−10^	7.03
21	**rs11154792**	6	135473333	T	C	MYB	Known	Single	**Erythrocytic**	7.45 × 10^−18^	17.89
22	**rs592423**	6	139882386	A	C	CITED2	Novel	Single	**Erythrocytic**	3.96 × 10^−8^	4.50
23	**rs2191349**	7	15030834	T	G	DGKB	Novel	Single	**Glycemic**	2.09 × 10^−7^	6.63
24	**rs4607517**	7	44202193	A	G	GCK	Known	Multiple	**Glycemic**	8.76 × 10^−38^	51.28
25	**rs3824065**	7	44213783	C	T	GCK	Novel	Multiple	**Glycemic**	4.22 × 10^−35^	31.87
26	**rs6474359**	8	41668351	T	C	ANK1	Known	Multiple	Unclassified	1.50 × 10^−16^	14.88
27	**rs4737009**	8	41749562	A	G	ANK1	Known	Multiple	**Erythrocytic**	4.48 × 10^−27^	32.08
28	**rs6980507**	8	42502241	A	G	SLC20A2	Novel	Single	**Erythrocytic**	3.58 × 10^−8^	8.73
29	**rs11558471**	8	118254914	A	G	SLC30A8	Known	Single	**Glycemic**	1.38 × 10^−19^	23.26
30	**rs2383208**	9	22122076	A	G	MTAP	Novel	Single	**Glycemic**	7.04 × 10^−12^	11.74
31	**rs7040409**	9	90693056	C	G	C9orf47	Novel	Single	**Erythrocytic**	2.56 × 10^−14^	11.29
32	**rs1467311**	9	109576753	G	A	KLF4	Novel	Single	Unclassified	2.09 × 10^−7^	8.72
33	**rs579459**	9	135143989	C	T	ABO	Novel	Single	**Glycemic**	9.42 × 10^−9^	10.14
34	**rs4745982**	10	70759849	T	G	HK1	Known	Multiple	**Erythrocytic**	2.87 × 10^−65^	63.05
35	**rs10823343**	10	70761019	A	G	HK1	Novel	Multiple	Unclassified	1.68 × 10^−55^	49.45
36	**rs17747324**	10	114742493	C	T	TCF7L2	Known	Single	**Glycemic**	6.12 × 10^−11^	8.49
37	**rs3782123**	11	195198	C	A	BET1L	Novel	Single	Unclassified	1.51 × 10^−10^	9.51
38	**rs2237896**	11	2815016	G	A	KCNQ1	Novel	Single	**Glycemic**	0.00246	6.07
39	**rs174577**	11	61361390	C	A	FADS2	Novel	Single	**Glycemic**	5.45 × 10^−7^	8.45
40	**rs11603334**	11	72110633	G	A	ARAP1	Novel	Single	**Glycemic**	6.85 × 10^−9^	6.53
41	**rs10830963**	11	92348358	G	C	MTNR1B	Known	Single	**Glycemic**	2.23 × 10^−23^	26.64
42	**rs11224302**	11	99961814	C	T	CNTN5	Novel	Single	**Erythrocytic**	4.76 × 10^−7^	6.40
43	**rs2110073**	12	6946143	T	C	PHB2	Novel	Single	Unclassified	4.44 × 10^−8^	7.18
44	**rs2408955**	12	46785398	T	G	SENP1	Novel	Single	**Erythrocytic**	1.42 × 10^−15^	11.65
45	**rs10774625**	12	110394602	G	A	ATXN2	Novel	Single	**Erythrocytic**	1.46 × 10^−8^	6.38
46	**rs11619319**	13	27385599	G	A	PDX1	Novel	Single	**Glycemic**	4.58 × 10^−7^	8.38
47	**rs576674**	13	32452302	G	A	KL	Novel	Single	**Glycemic**	1.39 × 10^−5^	6.38
48	**rs282587**	13	112399663	G	A	ATP11A	Known	Single	Unclassified	1.70 × 10^−12^	13.92
49	**rs9604573**	13	113571085	T	C	GAS6	Novel	Single	Unclassified	9.60 × 10^−9^	6.72
50	**rs11248914**	16	233563	T	C	ITFG3	Novel	Single	**Erythrocytic**	2.56 × 10^−14^	10.60
51	**rs1558902**	16	52361075	A	T	FTO	Novel	Single	Unclassified	3.27 × 10^−8^	6.88
52	**rs4783565**	16	67307691	A	G	CDH3	Novel	Single	**Erythrocytic**	1.73 × 10^−7^	6.73
53	**rs837763**	16	87381230	T	C	CDT1	Known	Single	**Erythrocytic**	1.68 × 10^−28^	28.89
54	**rs9914988**	17	24207230	A	G	ERAL1	Novel	Single	**Erythrocytic**	2.77 × 10^−11^	11.34
55	**rs2073285**	17	73628956	C	T	TMC6	Novel	Single	Unclassified	1.27 × 10^−4^	6.47
56	**rs1046896**	17	78278822	T	C	FN3KRP	Known	Single	Unclassified	4.46 × 10^−64^	71.79
57	**rs11086054**	19	17107737	A	T	MYO9B	Novel	Multiple	Unclassified	8.16 × 10^−6^	9.12
58	**rs17533903**	19	17117523	A	G	MYO9B	Known	Multiple	**Erythrocytic**	5.27 × 10^−12^	9.912
59	**rs4820268**	22	35799537	G	A	TMPRSS6	Known	Single	**Erythrocytic**	1.40 × 10^−22^	20.79
60	**rs1050828**	X	153417411	T	C	G6PD	Novel	Single	**Erythrocytic**	NA*	NA

*African American meta-analysis *p*-value for the *G6PD* variant (rs1050828) = 8.23 × 10^−135^. Chr, chromosome; MANTRA, Meta-Analysis of Transethnic Association

### Effect of HbA1c genetic scores on reclassification of prevalent undiagnosed T2D in population screening using HbA1c

Next, we tested whether erythrocytic variants influenced the ability of HbA1c to accurately classify individuals with diabetes when screening populations using a single HbA1c measurement. In 5 cohorts, among the 767 individuals with undiagnosed T2D by FG ≥ 7 mmol/L, 390 (50.8%) had measured HbA1c < 6.5% and would remain undiagnosed based on HbA1c. After accounting for the effect of erythrocytic variants, 5 (1.3%) of these individuals were correctly reclassified to having a HbA1c ≥ 6.5%. Among the 18,613 individuals without T2D by FG < 7 mmol/L, 266 (0.3%) had measured HbA1c ≥ 6.5% and would be incorrectly diagnosed with T2D by HbA1c. After accounting for the effect of erythrocytic variants, 50 (18.8%) of these individuals [13 of 80 (16.3%) European ancestry, 28 of 109 (25.7%) African ancestry, 9 of 77 (11.7%) Asian ancestry] were correctly reclassified to having a HbA1c<6.5% (Table 2, S6 Table). While adjusting for the effect of erythrocytic variants improved reclassification for individuals diagnosed with T2D by only HbA1c and not FG, it caused wrong reclassification for individuals diagnosed with T2D by both FG and HbA1c (S6 Table), suggesting that accounting for the contribution of erythrocytic variants may not be relevant for individuals who already meet diagnostic thresholds using both FG and HbA1c.

**Table 2:** Reclassification of individuals with discordant T2D status based on prevailing diagnostic thresholds for FG and HbA1c before and after accounting for the effect of erythrocytic variants.

	FG ≥ 7 mmol/L but HbA1c < 6.5% (*N* = 390)		FG < 7mmol/L but HbA1c ≥ 6.5% (*N* = 266)	
	Not reclassified after accounting for the effect of erythrocytic variants	Reclassified to HbA1c ≥ 6.5% after accounting for the effect of erythrocytic variants	Not reclassified after accounting for the effect of erythrocytic variants	Reclassified to HbA1c < 6.5% after accounting for the effect of erythrocytic variants
**European**	314	1 (0.3%)	67	13 (16.3%)
**African**	64	4 (6.3%)	81	28 (25.7%)
**Asian**	7	0 (0.0%)	68	9 (11.7%)
**Total**	385	5 (1.3%)	216	50 (18.8%)

FG, fasting glucose; HbA1c, glycated hemoglobin

### Effect of HbA1c genetic scores on prediction of incident T2D

**Fig 2:**
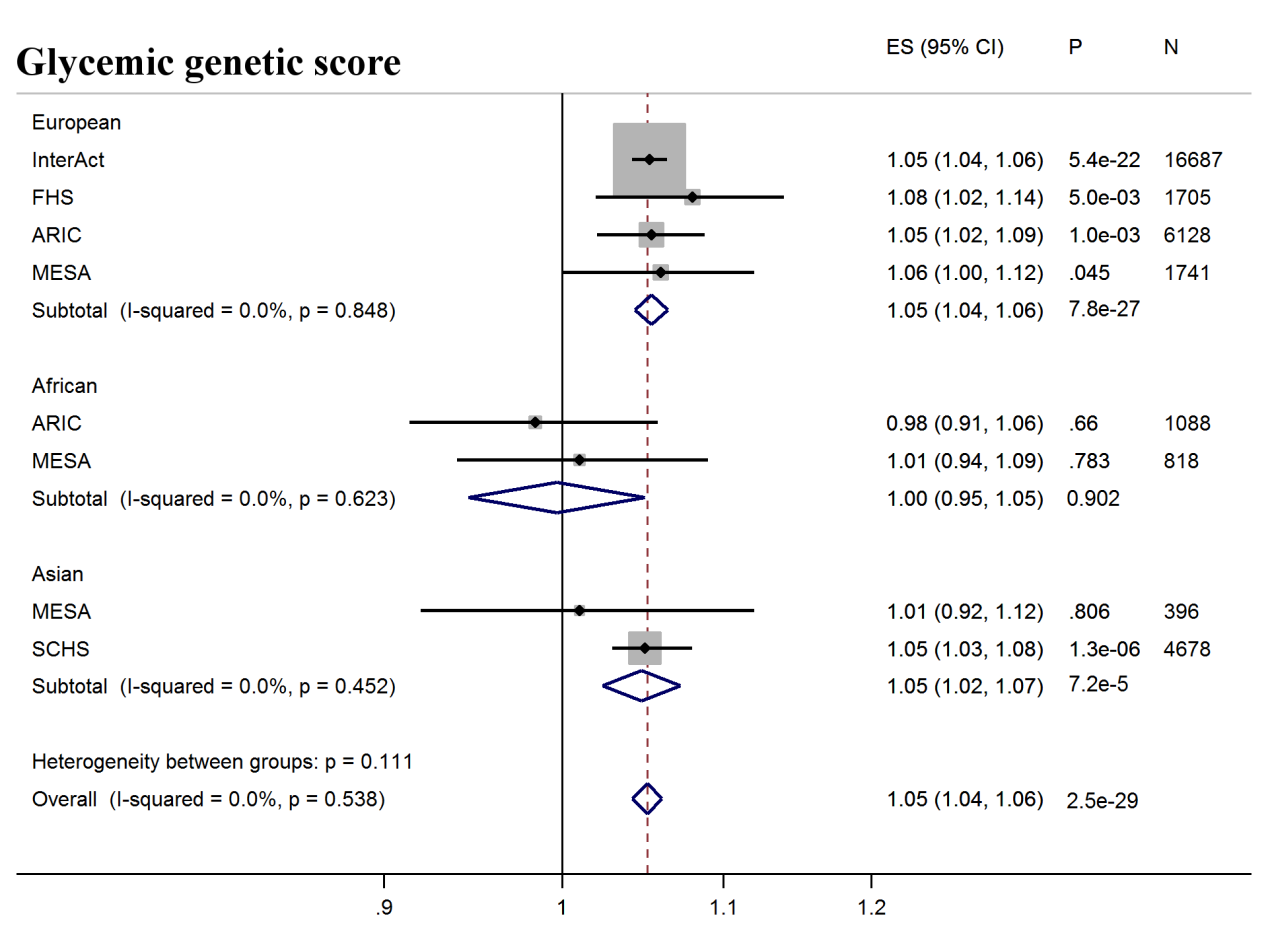
T2D prediction, glycemic genetic score. Forest plot of association between glycemic genetic score with incident T2D over a decade-long follow-up period, by ancestry. MESA (European and Asian ancestry) and the *G6PD* variant (rs1050828) in ARIC (European and African American) were not included in the discovery GWAS analysis. Effect estimates were combined in a fixed effects meta-analysis. Overall effect estimate: 1.05, 95% CI 1.04–1.06, *p* = 2.5 × 10^−29^. ARIC, Atherosclerosis Risk in Communities Study; ES, Effect Size; FHS, Framingham Heart Study; GWAS, genome-wide association study; G6PD, glucose-6-phosphate dehydrogenase; I-Squared, Higgin's I-squared statistic, a measure of heterogeneity; MESA, Multiethnic Study of Atherosclerosis; SCHS, Singapore Chinese Health Study; T2D, type 2 diabetes.

**Fig 3:**
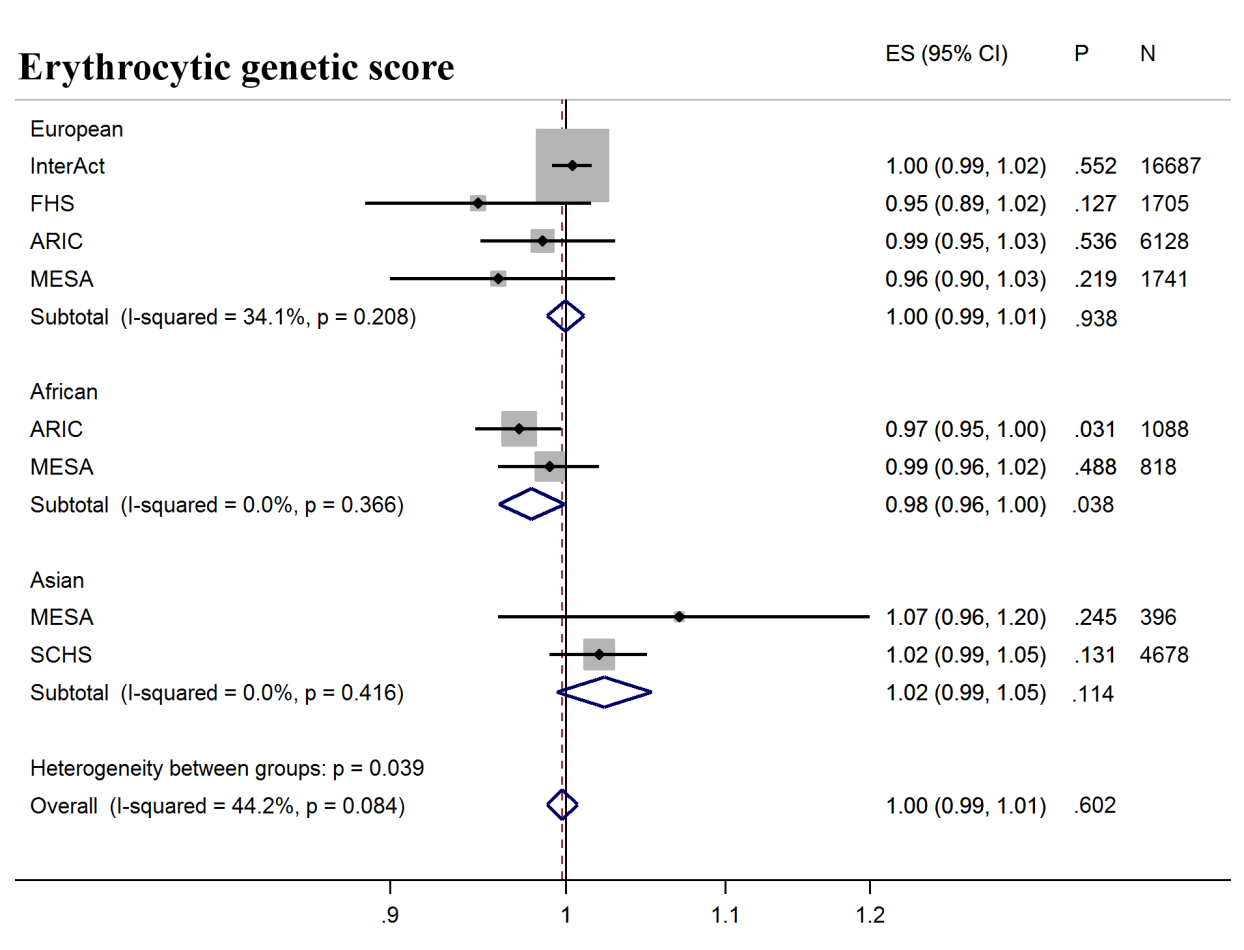
T2D prediction, erythrocytic genetic score. Forest plot of association between erythrocytic genetic score with incident T2D over a decade-long follow-up period, by ancestry. MESA (European and Asian ancestry) and the *G6PD* variant (rs1050828) in ARIC (European and African American) were not included in the discovery GWAS analysis. Effect estimates were combined in a fixed effects meta-analysis. Overall effect estimate: 1.00, 95% CI 0.99–1.01, *p* = 0.60. ARIC, Atherosclerosis Risk in Communities Study; ES, Effect Size, FHS, Framingham Heart Study; GWAS, genome-wide association study; G6PD, glucose-6-phosphate dehydrogenase; I-Squared, Higgin's I-squared statistic, a measure of heterogeneity; MESA, Multiethnic Study of Atherosclerosis; SCHS, Singapore Chinese Health Study; T2D, type 2 diabetes.

**Fig 4:**
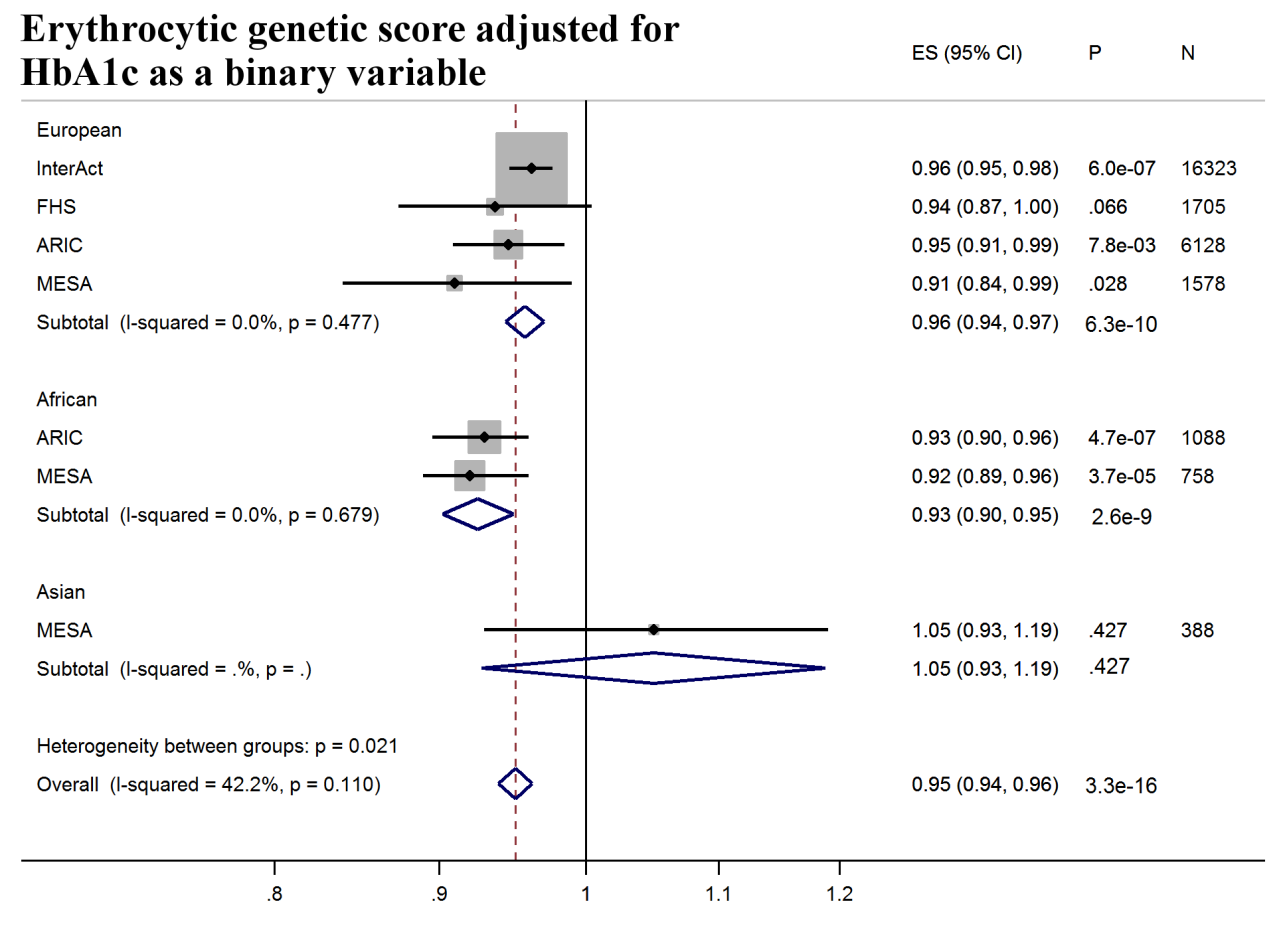
T2D prediction, erythrocytic genetic score adjusted for HbA1c as a binary variable. Forest plot of association between erythrocytic genetic score with incident T2D over a decade-long follow-up period adjusted for HbA1c as a binary variable (≥5.7% versus <5.7%), by ancestry. HbA1c at baseline was not available in SCHS and was excluded from the meta-analysis. MESA (European and Asian ancestry) and the *G6PD* variant (rs1050828) in ARIC (European and African American) were not included in the discovery GWAS analysis. Effect estimates were combined in a fixed effects meta-analysis. Overall effect estimate: 0.95, 95% CI 0.94–0.96, *p* = 3.3 × 10^−16^. ARIC, Atherosclerosis Risk in Communities Study; ES, Effect Size; GWAS, genome-wide association study; FHS, Framingham Heart Study; G6PD, glucose-6-phosphate dehydrogenase; HbA1c, glycated hemoglobin; I-Squared, Higgin's I-squared statistic, a measure of heterogeneity; MESA, multiethnic study of atherosclerosis; SCHS, Singapore Chinese Health Study; T2D, type 2 diabetes.

Next, we tested whether erythrocytic variants influenced the ability of HbA1c to predict incident diabetes in initially nondiabetic populations. GS-G was associated with increased incidence of T2D (odds ratio [OR] per weighted allele 1.05, 95% CI 1.04–1.06 *p* = 2.5 × 10^−29^) overall, although not in African Americans (Fig 2, S7 Table). GS-E was not associated overall with incident T2D (OR 1.00 95% CI 0.99–1.01, *p* = 0.60) (Fig 3, S7 Table), but was negatively associated with incident T2D in Europeans and African Americans after adjusting for HbA1c (OR 0.95, 95% CI, 0.94–0.96, *p* = 3.3 × 10^−16^) (Fig 4, S4 Fig and S7 Table), meaning individuals with a higher GS-E will have a lower risk of developing T2D given the same HbA1c value, suggesting that despite having the same HbA1c value, this does not reflect the same level of glycemia.

### Ancestral differences in the genetic architecture of HbA1c

**Fig 5:**
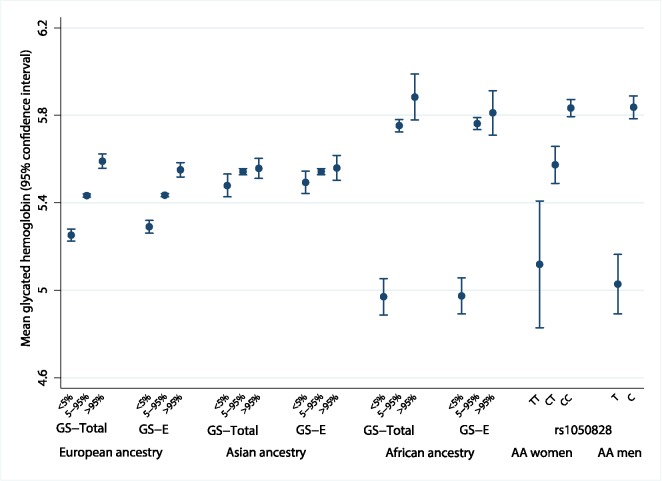
Mean HbA1c of individuals at the bottom 5% and top 5% of the distribution of ancestry-specific genetic scores and rs1050828 by genotype. The difference in measured HbA1c of individuals at the bottom 5% and top 5% of the distribution of an ancestry-specific additive GS composed of all 60 variants (GS-Total), and the equivalent calculation for an ancestry-specific GS composed of up to 20 erythrocytic variants (GS-E). Far right of the figure shows the mean HbA1c by genotype for chromosome X rs1050828. AA men, African American men; AA women, African American women; HbA1c, glycated hemoglobin; GS, genetic scores.

The population genetic history of African ancestry groups has undergone selective pressure due to the effects of malaria and other infectious diseases on erythrocytes, unlike in most European ancestry populations [35]. This led us to seek ancestral differences in the genetic determinants of HbA1c. The variance in HbA1c levels explained by all 60 genetic variants over a basic regression model including age and sex was 4.2%–5.8% in Europeans, 6.0%–14.3% in East Asians, and 8.9%–9.7% in African Americans (S8 Table). In addition, compared to Europeans and East Asians, African Americans had the largest difference in mean HbA1c between the bottom and top 5% of the GS-Total distribution (0.91%-units, 95% CI 0.78–1.05; Fig 5 and S9 Table).

Erythrocytic variants alone explained around one-fifth to three-quarters of the total explained genetic variance in HbA1c (S8 Table). The absolute differences in mean HbA1c from the bottom and top 5% of the GS-E distribution were similar to GS-Total, implying that genetically-induced differences in HbA1c may be largely driven by erythrocytic variants (S9 Table and S10 Table). In African Americans, this difference was largely driven by the C-to-T missense variant (G202A) in *G6PD*, rs1050828 on chromosome X. This variant alone explained 14.4% of variance in HbA1c (MESA; 9.6% in women; 19.9% in men). Men with the T allele had, on average, an absolute 0.81%-units (95% CI 0.66–0.96) lower HbA1c than those with the C allele. Homozygous TT women had, on average, an absolute 0.68%-units (95% CI 0.38–0.97) lower HbA1c compared to CC homozygous women. The effect size was similar after excluding those with anemia (Hb < 12 g/dL in women and < 13 g/dL in men, S11 Table).

Fructosamine is another measure of serum protein glycation, which reflects glycemia over a 2–3 week window, but unlike HbA1c it is not influenced by RBC traits; therefore, we sought to explore the difference between fructosamine-inferred HbA1c and measured HbA1c (Methods, S1 Analysis Plans) to test the hypothesis that the *G6PD* variant might be influencing HbA1c levels independently of ambient glycemia. Among African Americans, the T allele at rs1050828 was associated with measured HbA1c that was lower than fructosamine-predicted HbA1c (0.31%-units, 95% CI 0.25–0.37, *p* = 6.4 × 10^−19^). Among men with the C allele, measured HbA1c was similar to fructosamine-predicted HbA1c (residuals, 0.04%-units, 95% CI −0.04 to 0.12, *N* = 351). This suggested that only the T allele was associated with markedly lower HbA1c than expected from glycemic measurements (S11 Table).

### Public health implications of the *G6PD* variant on T2D screening

Given the large effects of the *G6PD* G202A variant on HbA1c levels, we sought to investigate the impact this variant would have on diabetes detection if using HbA1c as a screening tool. To do this, we used publicly-available data from NHANES 2013–2014 [34], a nationally representative sample of the US, to calculate the proportion of African Americans adults with T2D who would be missed by not accounting for rs1050828 when using a single HbA1c diagnostic threshold of 6.5%, assuming the observed effect size of rs1050828, allele frequency of 11%, and accounting for NHANES sampling design. In the NHANES sample of African Americans (*N* = 1,133), the mean age was 44.2 years (standard error 0.9), 55.2% were women, and mean HbA1c, excluding those with physician-diagnosed T2D, was 5.5%-units (standard error 0.02). 13.45% of African American adults aged ≥ 18 years had physician-diagnosed T2D with an additional 2.50% with undiagnosed T2D by HbA1c ≥ 6.5%. An additional estimated 2.17% (95% CI 1.88–2.46) with HbA1c < 6.5% may be considered to have T2D if the effect of rs1050828 was accounted for by using genotype-specific diagnostic thresholds of 5.7% for T in men, 5.8% for TT, and 6.2% for TC in women. According to the 2014 United States Census Bureau, approximately 29.9 million adults identified themselves as African American [36], suggesting that 0.65 (95% CI 0.55–0.74) million adults with T2D would remain undiagnosed when screened by a single HbA1c measurement if this genetic information were not taken into account (S12 Table).

## Discussion

In a very large transancestry GWAS of HbA1c, we identified 42 novel and 18 known genetic variants associated with HbA1c, explaining 4%–14% of the trait variance. Genetic variants influencing HbA1c through erythrocytic pathways did not predict future T2D, and adjusting for their contribution to HbA1c led to a moderate misclassification of T2D by adjusted HbA1c. Notably, we detected strong ancestral differences in the contribution of genetic variants to HbA1c that substantially altered the performance of HbA1c as a diagnostic test for T2D in African Americans compared with Europeans and East Asians.

Our findings elucidate the contribution of common genetic variants to the genetic architecture of HbA1c and identify an important interface of modern human genetics with clinical and public health. In people of European and Asian ancestry, we found multiple genetic loci with small-to-modest effects, whereas, in African American ancestry, the genetic architecture was dominated by a single variant at *G6PD* (G202A). This variant was responsible for 0.81%-units HbA1c difference in men and 0.68%-units in homozygous TT women, corresponding to adjusted T2D diagnosis thresholds of 5.7 (95% CI 5.5–5.8) and 5.8 (95% CI 5.5–6.1), respectively. To meet the NGSP certification criteria, laboratory-reported HbA1c ought to be within 6% of the standard reference laboratory mean values (e.g., 6.5%-units ± 0.4%-units) for the majority of patient samples [14]. The limits of acceptable analytic variability were exceeded by this *G6PD* variant. This may also have important implications for the management of diabetes, with carriers of the HbA1c-lowering *G6PD* allele requiring adjusted (lower) HbA1c treatment targets. Previous epidemiologic studies have shown that a 1%-unit increase in HbA1c in individuals without T2D was associated with a more than 2-fold increase in risk of future T2D and a 20%–50% increased risk of cardiovascular disease (CVD) [37]. HbA1c ≥ 6.5% compared to those with HbA1c < 5.7% had a higher risk of kidney disease and retinopathy [38].

Only one other African-specific variant, rs11954649, located in the intron of *SOX30*, reached genome-wide significance in African Americans. However, this variant had a relatively small effect size (β = 0.12 per G allele) on HbA1c and was not classified as glycemic or erythrocytic. The variant was thus not included in the genetic scores and, unlike *G6PD*, the causal transcript and biological mechanism through which it influences HbA1c remains unclear. Future studies on larger sample sizes of ethnic minorities can focus on dissecting the genomic and biological implications of novel HbA1c-related variants.

When considering all ethnicities, both glycemic and erythrocytic variants influence measured HbA1c; yet, only glycemic variants were associated with increased T2D risk (5% per allele) over a decade-long follow-up period. For an equivalent HbA1c, individuals carrying more erythrocytic HbA1c-raising alleles, or fewer HbA1c-lowering alleles, had lower incident T2D risk (−5% per allele), implying that for the same HbA1c level those individuals with the greater number of erythrocytic HbA1c- raising alleles have artificially higher HbA1c values that do not reflect ambient glycemia. Thus, the influence of erythrocytic HbA1c variants may partly explain why some individuals with the same HbA1c may have different risks of future T2D. We note that the estimates of variance explained by genetic variants underlying HbA1c were comparable with those for FG in Europeans (4.8%) [17].

Our results on the reclassification of prevalent T2D were consistent with previous reports indicating that a diagnostic cut-point at 6.5% for HbA1c classified fewer cases than FG ≥ 7 mmol/L [39,40]. Adjusting for the contribution of erythrocytic variants correctly reclassified approximately 1 in 5 individuals with FG < 7 mmol/L who were incorrectly diagnosed as having T2D (HbA1c ≥ 6.5%) to having HbA1c < 6.5%, suggesting that a subset of these individuals may have artificially elevated HbA1c due to the contribution of the erythrocytic variants.

Though the specific *G6PD* variant we identified is monomorphic in Asian and European ancestry, other diverse *G6PD* variant alleles have reached polymorphic frequencies in malarial endemic regions around the world [35]. G6PD deficiency is unlikely to be identified through routine screening for anemia in healthy individuals, and universal screening for G6PD deficiency is not currently recommended worldwide [32,41]. Testing for G6PD deficiency is only performed on individuals before being prescribed specific drugs, such an antimalarial medications, or in patients with clinical presentation consistent with the disease; for instance, prolonged neonatal jaundice or hemolytic crisis following exposure to specific drugs, infections, or foods [32]. Thus, asymptomatic individuals often remain unaware of their *G6PD* genotype status and screening for the *G6PD* genotype before using HbA1c to diagnose T2D may be warranted in populations or ethnic groups where G6PD deficiency is common. Similarly, a recent study identified a significant hemolytic risk in women heterozygous for the *G6PD* Mahidol variant when treated with primaquine who were not detected by current screening methods [42]. Rarer hematologic conditions that reduce erythrocyte lifespan, e.g., hereditary hemolytic anemias, hereditary spherocytosis, and hemoglobinopathies have also been shown to lower HbA1c [9,43], and should also be considered before using HbA1c in these patients. We recommend additional testing using direct glucose measurements (e.g., FG or oral glucose tolerance testing) or other erythrocyte-independent methods to diagnose T2D. This supports the use of a combination of HbA1c and FG to confirm T2D diagnosis in routine screening [44]. Future studies could also explore *G6PD* effect modification by HbA1c assay type.

Further studies in large cohorts with HbA1c, glycemic, and erythrocytic traits are required to better determine the biological action of genetic variants that have yet to be classified. Similarly, future analyses conditional on RBC distribution width or reticulocyte count will help to better understand the effects of erythrocytic HbA1c-associated variants, should such data become available. The relatively small sample size for Asian and African ancestry cohorts limited the discovery of ancestry-specific genetic variants, beyond the African-specific *G6PD* variant, and could explain why GS-G was associated with higher incident T2D in European, but not other, ancestries. This underscores the need to extend such studies to non-European populations, particularly those with a high prevalence of some hemoglobinopathies or iron deficiency anemias. Epidemiologic studies have reported higher mean HbA1c in African Americans compared to European ancestry individuals in the US [45,46]. While our genetic findings could not determine whether this difference was completely attributable to relative hyperglycemia, accounting for the effect of the *G6PD* variant that lowers HbA1c only in African Americans would further widen this disparity.

In conclusion, HbA1c remains an appropriate diagnostic test for the majority of people of diverse genetic backgrounds, having lower intraindividual variability compared to FG with the ability to capture chronic hyperglycemia, and robust associations with T2D-related complications [37]. Nevertheless, nonglycemic lowering of measured HbA1c for 1 in 10 African American men who carry this *G6PD* variant, and 1 in a 100 African American women homozygous for this variant, could amount to 0.65 (95% CI 0.56–0.74) million African American adults in the US with a missed T2D diagnosis using HbA1c as a screening test. We therefore recommend investigation of the possible benefits of screening for the *G6PD* genotype along with using HbA1c to diagnose T2D in populations of African ancestry or groups where G6PD deficiency is common, and screening with direct glucose measurements, or genetically-informed HbA1c diagnostic thresholds in people with G6PD deficiency. This work supports a role for a precision medicine application to reduce race-ethnic health disparities using HbA1c genetics to improve T2D diagnosis and prediction and to inform screening strategies for T2D across the African continent where the prevalence of the *G6PD* variant can reach 20%.

## Supporting information

S1 Checklist STREGA checklist. (DOC)

S1 Table Cohort information, genotyping, quality control (QC), glycated hemoglobin (HbA1c), analysis and covariates. (XLSX)

S2 Table Association of lead glycated hemoglobin (HbA1c) variants with glycemic and erythrocytic traits from publicly available association results. (XLSX)

S3 Table Attenuation of glycated hemoglobin variant (HbA1c) effect size in association models conditioned on fasting glucose (FG), 2hr glucose (2hrGlu), hemoglobin level (Hb), mean corpuscular volume (MCV), and mean corpuscular hemoglobin (MCH) for the lead HbA1c-associated variants. (XLSX)

S4 Table Baseline characteristics among those who developed incident type 2 diabetes (T2D) during follow-up and among those who did not, by cohort and ethnicity. (XLSX)

S5 Table Genome-wide significant SNPs identified in the genetic discovery analysis. (XLSX)

S6 Table Net reclassification index of type 2 diabetes (T2D) status by measured glycated hemoglobin (HbA1c) ≥ 6.5% compared to fasting glucose (FG) ≥ 7 mmol/L with and without accounting for erythrocytic genetic variants by ancestry. (XLSX)

S7 Table Association of genetic score-glycemic (GS-G) and genetic score erythrocytic (GS-E) with incident type 2 diabetes (T2D) over a decade-long follow-up period by cohort and ancestry. (XLSX)

S8 Table Proportion of additional variance explained over age and sex in measured glycated hemoglobin (HbA1c) by erythryocytic genetic variants and by all genome-wide significant genetic variants by ethnicity. (XLSX)

S9 Table Mean difference in glycated hemoglobin (HbA1c) between the top and bottom 5 percentile of genetic score-total (GS-Total) by ancestry. (XLSX)

S10 Table Mean difference in glycated hemoglobin (HbA1c) between the top and bottom 5 percentile of genetic score-erythrocytic (GS-E) by ancestry. (XLSX)

S11 Table Additional analyses on the association of rs1050828, *G6PD* variant G202A, with glycated hemoglobin (HbA1c). (XLSX)

S12 Table Estimated number of African Americans with type 2 diabetes (T2D) in the US whose diagnosis would be missed due to the glycose-6-phosphate dehydrogenase (*G6PD*) variant if screened with glycated hemoglobin (HbA1c). (XLSX)

S1 Fig Diagram describing the flow of our study. (PDF)

S2 Fig Overview of participants included in the genetic discovery analysis. (PDF)

S3 Fig Overview of the classification of genetic variants as glycemic, erythrocytic, or unclassified. (PDF)

S4 Fig Forest plot of association between erythrocytic genetic score with incident type 2 diabetes (T2D) over a decade-long follow-up period adjusted for glycated hemoglobin (HbA1c) as a continuous variable by ancestry. (PDF)

S1 Analysis Plans Analysis plans. (DOCX)

S1 Financial Disclosure Authors’ funding information. (DOCX)

## Abbreviations

ARIC: Atherosclerosis Risk in Communities Study
CVD: cardiovascular disease
EPIC-InterAct: European Prospective Investigation into Cancer and Nutrition InterAct project
FG: fasting glucose
FHS: Framingham Heart Study
GCTA: Genome-wide Complex Trait Analysis
GS-E: genetic scores of erythrocytic variants
GS-G: genetics scores of glycemic variants
G6PD: glucose-6-phosphate dehydrogenase
GWAS: genome-wide association studies
Hb: hemoglobin level
HbA1c: glycated hemoglobin
JHS: Jackson Heart Study
LD: linkage disequilibrium
LOLIPOP: London Life Sciences Prospective Population Study
MAF: minor allele frequency
MANTRA: Meta-Analysis of Transethnic Association
MCH: mean corpuscular hemoglobin
MCV: mean corpuscular volume
MESA: Multiethnic Study of Atherosclerosis
NGSP: National Glycohemoglobin Standardization Program
NHANES: National Health and Nutrition Examination Survey
OR: odds ratio
QC: quality control
RBC: red blood cell
SCHS: Singapore Chinese Health Study
SiMES: Singapore Malay Eye Study
SP2: Singapore Prospective Study
TAICHI: Taiwan-Metabochip Study for Cardiovascular Disease
T2D: type 2 diabetes
WGHS: Women’s Genome Health Study
2hrGlu: 2hr glucose
