# Supplementary material for: Impact of common genetic determinants of Hemoglobin A1c on type 2 diabetes risk and diagnosis in ancestrally diverse populations: A transethnic genome-wide meta-analysis
Source: PLoS Med. 2017 Sep 12;14(9):e1002383. doi: 10.1371/journal.pmed.1002383 (PMC5595282; doi:10.1371/journal.pmed.1002383)
Supplement: S1 Checklist — (DOC) [file pmed.1002383.s001.doc]

# S1 Checklist: STREGA reporting recommendations, extended from STROBE Statement

| **Item** | **Item number** | **STROBE Guideline** | | **Extension for Genetic Association Studies (STREGA)** | **Relevant text from manuscript** | |
| --- | --- | --- | --- | --- | --- | --- |
| **Title and Abstract** | 1 | (a) Indicate the study’s design with a commonly used term in the title or the abstract. | |  | Abstract:  “genome-wide association meta-analyses” | |
|  |  | (b) Provide in the abstract an informative and balanced summary of what was done and what was found. | |  | Abstract | |
| **Introduction** | | |  | | |  |
| *Background rationale* | 2 | Explain the scientific background and rationale for the investigation being reported. | |  | Abstract, background  Introduction | |
| *Objectives* | 3 | State specific objectives, including any pre-specified hypotheses. | | ***State if the study is the first report of a genetic association, a replication effort, or both.*** | Abstract, background  Introduction | |
| **Methods** | | |  | | |  |
| *Study design* | 4 | Present key elements of study design early in the paper. | |  | Introduction, paragraph 3  Supplementary Figure 1, S1 Appendix | |
| *Setting* | 5 | Describe the setting, locations and relevant dates, including periods of recruitment, exposure, follow-up, and data collection. | |  | All relevant cohort information is presented in S1 Table | |
| *Participants* | 6 | 1. **Cohort study –** Give the eligibility criteria, and the sources and methods of selection of participants. Describe methods of follow-up.   **Case-control study –** Give the eligibility criteria, and the sources and methods of case ascertainment and control selection. Give the rationale for the choice of cases and controls.  **Cross-sectional study –** Give the eligibility criteria, and the sources and methods of selection of participants. | | ***Give information on the criteria and methods for selection of subsets of participants from a larger study, when relevant***. | All relevant cohort information including sample exclusions are presented in S1 Table. Analysis plans with exclusion criteria for the discovery genetic analysis are included in S1 Analysis Plans | |
|  |  | 1. **Cohort study –** For matched studies, give matching criteria and number of exposed and unexposed.   **Case-control study –** For matched studies, give matching criteria and the number of controls per case. | |  |  | |
| *Variables* | 7 | *(a)* Clearly define all outcomes, exposures, predictors, potential confounders, and effect modifiers. Give diagnostic criteria, if applicable. | | ***(b)*** ***Clearly define genetic exposures (genetic variants) using a widely-used nomenclature system. Identify variables likely to be associated with population stratification (confounding by ethnic origin).*** | Genetic variants identified in the discovery meta-analysis are presented in S2 Table. Classification of variants included in the glycemic and erythrocytic risk scores are presented in S2 Table.  Prevalent T2D: Methods, Effect of HbA1c Genetic Scores on Reclassification of Prevalent Undiagnosed T2D for Population Screening using HbA1c  Incident T2D: Methods, Effect of HbA1c Genetic Scores on Prediction of Incident T2D | |
| *Data sources measurement* | 8***** | *(a)* For each variable of interest, give sources of data and details of methods of assessment (measurement). Describe comparability of assessment methods if there is more than one group. | | ***(b)*** ***Describe laboratory methods, including source and storage of DNA, genotyping methods and platforms (including the allele calling algorithm used, and its version), error rates and call rates. State the laboratory/centre where genotyping was done****.* ***Describe comparability of laboratory methods if there is more than one group. Specify whether genotypes were assigned using all of the data from the study simultaneously or in smaller batches.*** | S1 Table | |
| *Bias* | 9 | *(a)* Describe any efforts to address potential sources of bias. | | ***(b) For quantitative outcome variables, specify if any investigation of potential bias resulting from pharmacotherapy was undertaken. If relevant, describe the nature and magnitude of the potential bias, and explain what approach was used to deal with this.*** | Not applicable | |
| *Study size* | 10 | Explain how the study size was arrived at. | |  | Not applicable, retrospective study | |
| *Quantitative variables* | 11 | Explain how quantitative variables were handled in the analyses. If applicable, describe which groupings were chosen, and why. | | ***If applicable, describe how effects of treatment were dealt with.*** | S1 Table  Methods | |
| Statistical methods | 12 | (a) Describe all statistical methods, including those used to control for confounding. | | ***State software version used and options (or settings) chosen.*** | Methods  S1 Table  S1 Analysis Plans | |
|  |  | (b) Describe any methods used to examine subgroups and interactions. | |  | Not applicable | |
|  |  | (c) Explain how missing data were addressed. | |  | Imputation of genetic variants not directly genotyped. Methods, Genotyping and Quality Control | |
|  |  | 1. **Cohort study –** If applicable, explain how loss to follow-up was addressed.   **Case-control study –** If applicable, explain how matching of cases and controls was addressed.  **Cross-sectional study –** If applicable, describe analytical methods taking account of sampling strategy. | |  | NHANES analysis: Methods, Estimated number of African Americans with T2D in United States whose diagnosis would be missed due to the G6PD variant if screened with glycated hemoglobin | |
|  |  | (e) Describe any sensitivity analyses. | |  | Not applicable | |
|  |  |  | | ***(f) State whether Hardy-Weinberg equilibrium was considered and, if so, how****.* | Methods, Genotyping and Quality Control  S1 Table | |
|  |  |  | | ***(g) Describe any methods used for inferring genotypes or haplotypes.*** | Methods, Genotyping and Quality Control  S1 Table | |
|  |  |  | | ***(h) Describe any methods used to assess or address population stratification.*** | Methods, Genetic Discovery using Ancestry-Specific and Trans-Ancestry Meta-Analyses | |
|  |  |  | | ***(i) Describe any methods used to address multiple comparisons or to control risk of false positive findings.*** | Methods, Identification of Primary and Secondary Distinct HbA1c-Associated Signals  “based on P=0.05 divided by the estimated number of independent tests across the genome” | |
|  |  |  | | ***(j) Describe any methods used to address and correct for relatedness among subjects*** | S1 Table | |
| **Results** | | |  | | |  |
| *Participants* | 13***** | 1. Report the numbers of individuals at each stage of the study – e.g., numbers potentially eligible, examined for eligibility, confirmed eligible, included in the study, completing follow-up, and analysed. | | ***Report numbers of individuals in whom genotyping was attempted and numbers of individuals in whom genotyping was successful.*** | S1 Table | |
|  |  | (b) Give reasons for non-participation at each stage. | |  | Not applicable | |
|  |  | (c) Consider use of a flow diagram. | |  | Not applicable | |
| *Descriptive data* | 14***** | (a) Give characteristics of study participants (e.g., demographic, clinical, social) and information on exposures and potential confounders. | | ***Consider giving information by genotype****.* | S1 Table | |
|  |  | (b) Indicate the number of participants with missing data for each variable of interest. | |  | Maximum number of participants for any SNP analysed reported in S1 Table | |
|  |  | 1. **Cohort study –** Summarize follow-up time, e.g. average and total amount. | |  | Not applicable | |
| *Outcome data* | 15 ***** | **Cohort study-**Report numbers of outcome events or summary measures over time. | | ***Report outcomes (phenotypes) for each genotype category over time*** |  | |
|  |  | **Case-control study –** Report numbers in each exposure category, or summary measures of exposure. | | ***Report numbers in each genotype category*** |  | |
|  |  | **Cross-sectional study –** Report numbers of outcome events or summary measures. | | ***Report outcomes (phenotypes) for each genotype category*** | Effect estimates for each SNP on HbA1c are presented for each ancestry in S2 Table | |
| *Main results* | 16 | (a) Give unadjusted estimates and, if applicable, confounder-adjusted estimates and their precision (e.g., 95% confidence intervals). Make clear which confounders were adjusted for and why they were included. | |  | S2 Table | |
|  |  | (b) Report category boundaries when continuous variables were categorized. | |  | Results, Effect of HbA1c Genetic Scores on Reclassification of Prevalent Undiagnosed T2D in Population Screening using HbA1c  Results, Effect of HbA1c Genetic Scores on Prediction of Incident T2D | |
|  |  | (c) If relevant, consider translating estimates of relative risk into absolute risk for a meaningful time period. | |  | Not applicable | |
|  |  |  | | ***(d) Report results of any adjustments for multiple comparisons.*** | Not applicable, threshold for genetic discovery analysis is based on the number of independent tests | |
| *Other analyses* | 17 | 1. Report other analyses done – e.g., analyses of subgroups and interactions, and sensitivity analyses. | |  | Not applicable | |
|  |  |  | | ***(b) If numerous genetic exposures (genetic variants) were examined, summarize results from all analyses undertaken.*** | Results, HbA1c-associated Genetic Variants and Classification by Glycemic and Non-Glycemic Pathways  Fig 1. HbA1c associated variants | |
|  |  |  | | ***(c) If detailed results are available elsewhere, state how they can be accessed.*** | Data availability | |
| **Discussion** | | |  | | |  |
| *Key results* | 18 | Summarize key results with reference to study objectives. | |  | Discussion, paragraphs 1,4,5 and 8 | |
| *Limitations* | 19 | Discuss limitations of the study, taking into account sources of potential bias or imprecision. Discuss both direction and magnitude of any potential bias. | |  | Abstract  Introduction, Methods & Findings  Discussion, paragraphs 3 and 7 | |
| *Interpretation* | 20 | Give a cautious overall interpretation of results considering objectives, limitations, multiplicity of analyses, results from similar studies, and other relevant evidence. | |  | Discussion, paragraph 8 | |
| *Generalizability* | 21 | Discuss the generalizability (external validity) of the study results. | |  | Discussion | |
| **Other Information** | | |  | | |  |
| *Funding* | 22 | Give the source of funding and the role of the funders for the present study and, if applicable, for the original study on which the present article is based. | |  | S1 Appendix, Personal and Study Acknowledgements | |

STREGA = STrengthening the REporting of Genetic Association studies; STROBE = STtrengthening the Reporting of Observational Studies in Epidemiology.
