# Supplementary figures and images for: Impact of common genetic determinants of Hemoglobin A1c on type 2 diabetes risk and diagnosis in ancestrally diverse populations: A transethnic genome-wide meta-analysis
Source: PLoS Med. 2017 Sep 12;14(9):e1002383. doi: 10.1371/journal.pmed.1002383 (PMC5595282; doi:10.1371/journal.pmed.1002383)

**S1 Fig.** Diagram describing the flow of our study

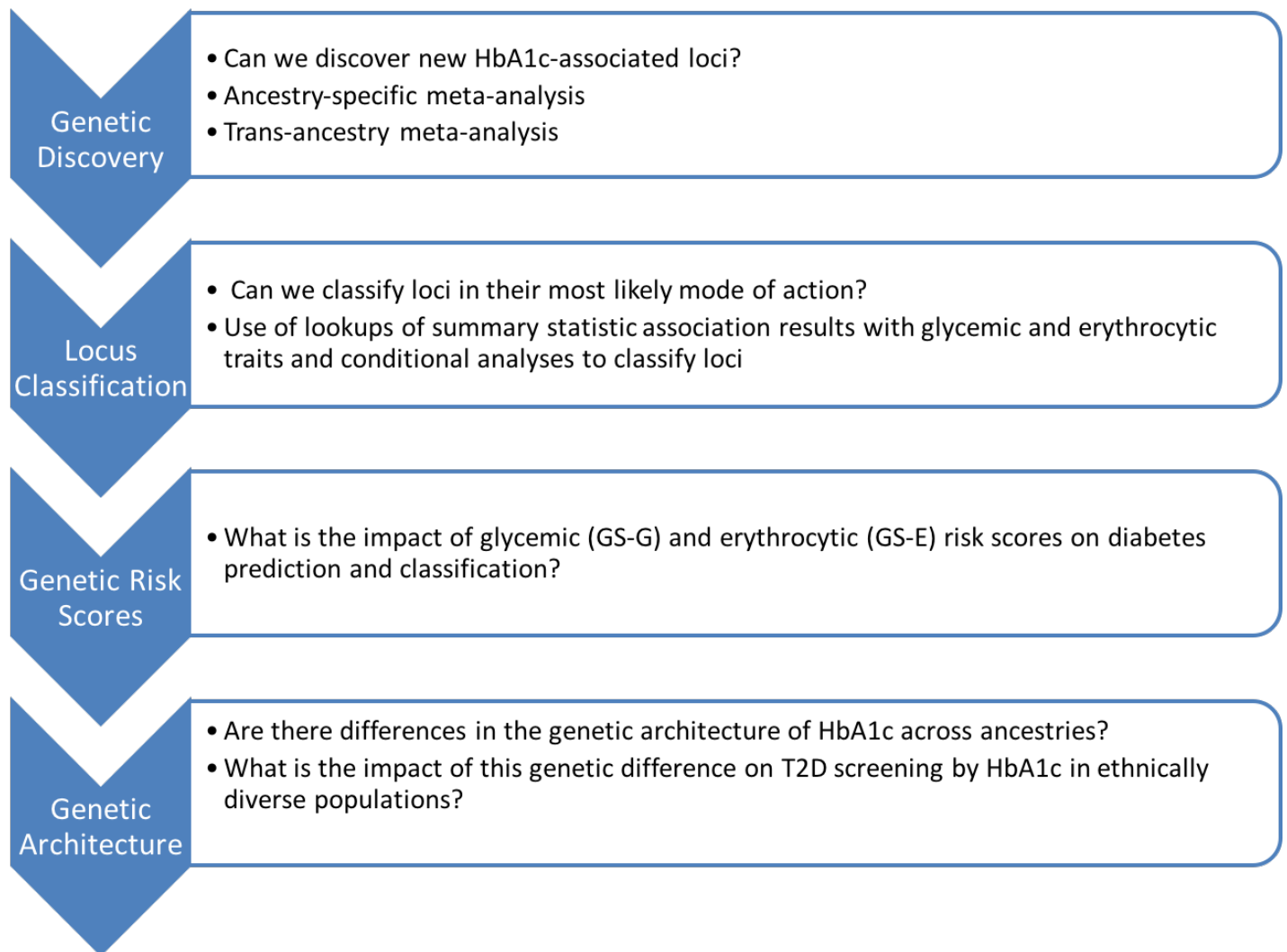

Supplement: S1 Fig — (PDF) [file pmed.1002383.s014.pdf]
