# Supplementary material for: Impact of common genetic determinants of Hemoglobin A1c on type 2 diabetes risk and diagnosis in ancestrally diverse populations: A transethnic genome-wide meta-analysis
Source: PLoS Med. 2017 Sep 12;14(9):e1002383. doi: 10.1371/journal.pmed.1002383 (PMC5595282; doi:10.1371/journal.pmed.1002383)
Supplement: S2 Fig — (PDF) [file pmed.1002383.s015.pdf]

S2 Fig. Overview of participants included in the genetic discovery analysis.

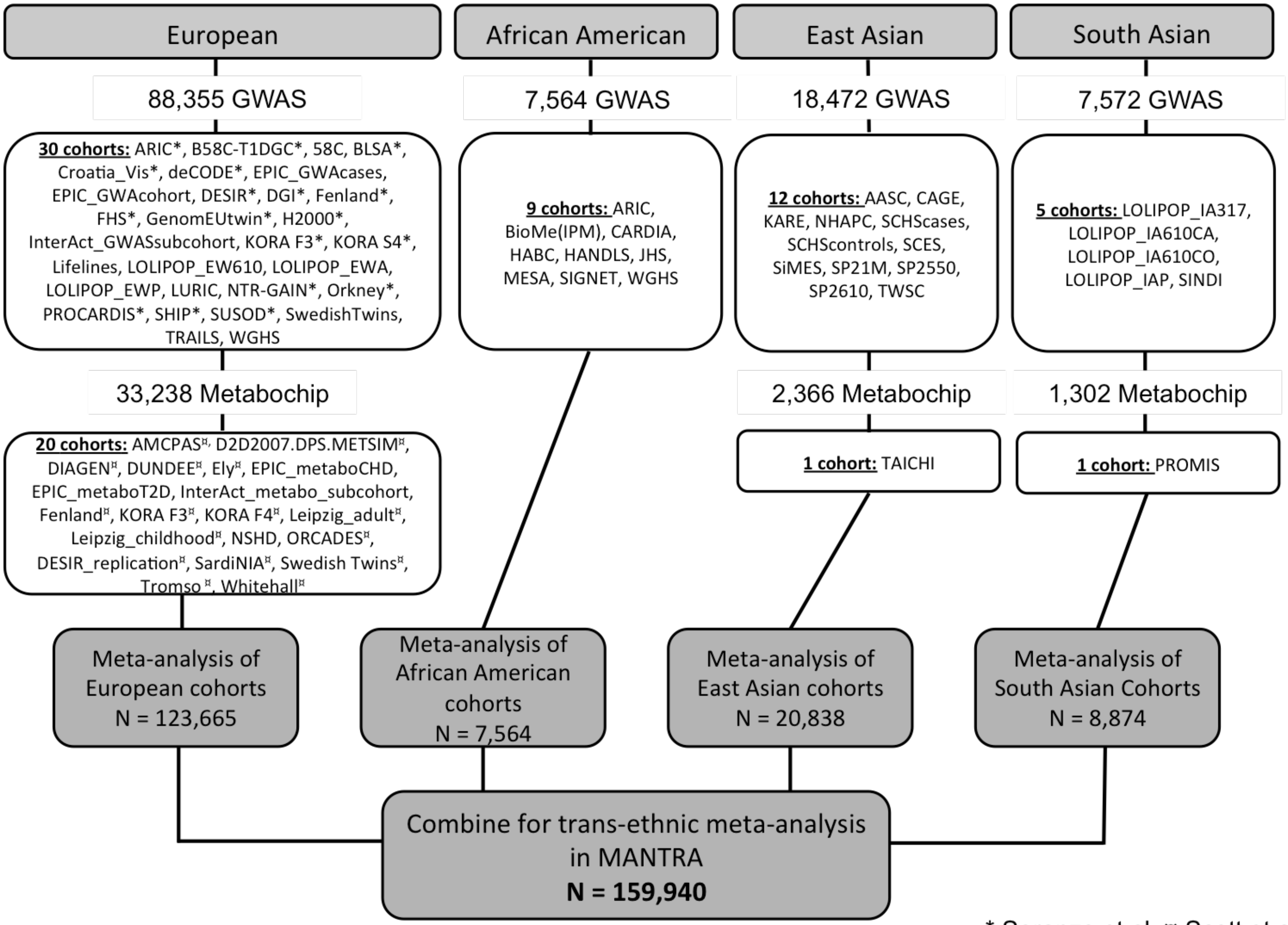

* Soranzo et al, ¤ Scott et al
