# Supplementary material for: Impact of common genetic determinants of Hemoglobin A1c on type 2 diabetes risk and diagnosis in ancestrally diverse populations: A transethnic genome-wide meta-analysis
Source: PLoS Med. 2017 Sep 12;14(9):e1002383. doi: 10.1371/journal.pmed.1002383 (PMC5595282; doi:10.1371/journal.pmed.1002383)
Supplement: S3 Fig — (PDF) [file pmed.1002383.s016.pdf]

S3 Fig. Overview of the classification of genetic variants as glycemic, erythrocytic or unclassified.

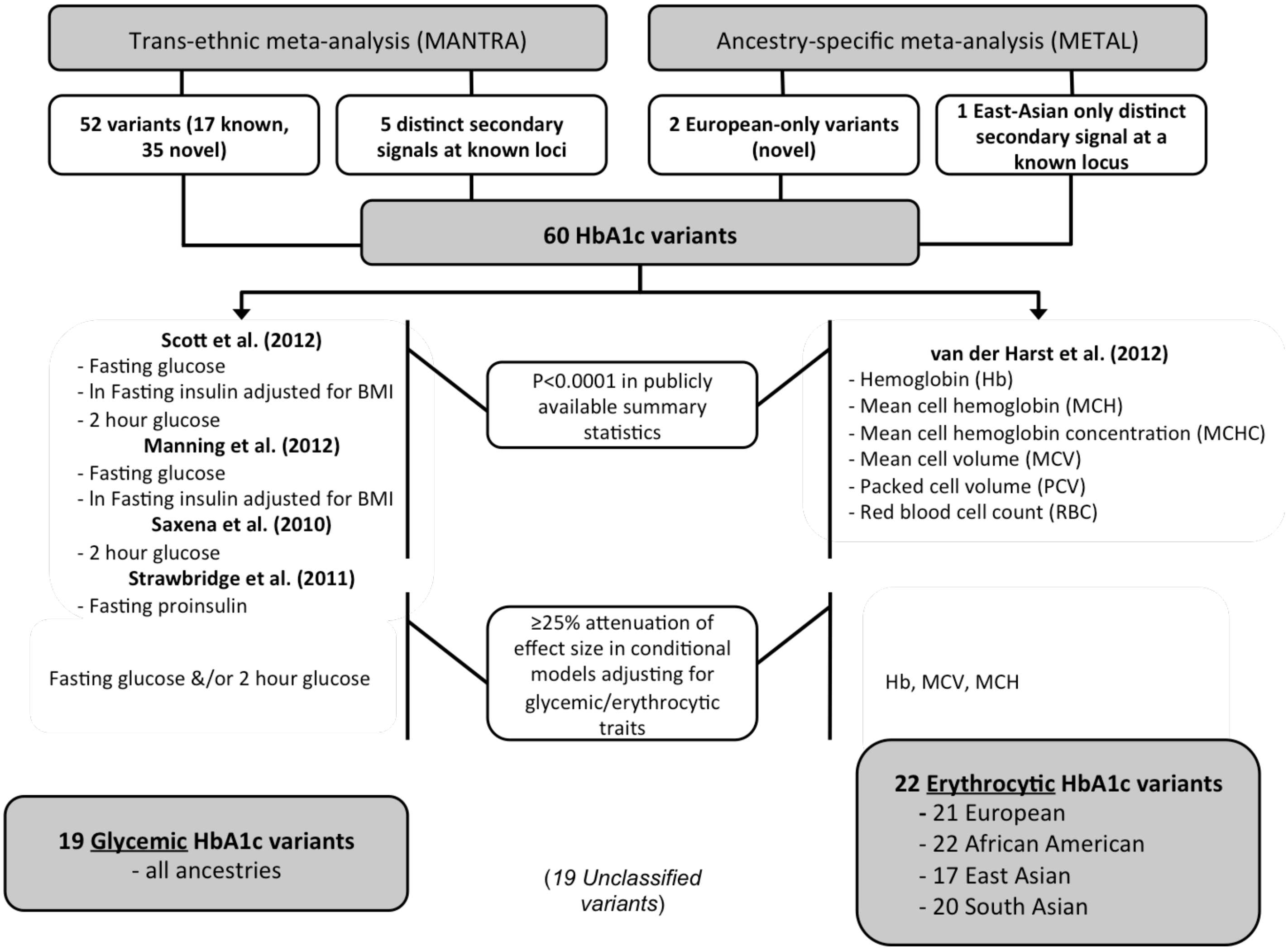

A single variant (rs579459 near *ABO*) was classified as both glycemic and erythrocytic, but treated as glycemic in all analyses.
