# Supplementary material for: Impact of common genetic determinants of Hemoglobin A1c on type 2 diabetes risk and diagnosis in ancestrally diverse populations: A transethnic genome-wide meta-analysis
Source: PLoS Med. 2017 Sep 12;14(9):e1002383. doi: 10.1371/journal.pmed.1002383 (PMC5595282; doi:10.1371/journal.pmed.1002383)

S4 Fig. Forest plot of association between erythrocytic genetic score with incident type 2 diabetes (T2D) over a decade-long follow-up period adjusted for glycated hemoglobin (HbA1c) as a continuous variable by ancestry.

Glycated hemoglobin at baseline was not available in SCHS and was excluded from the meta-analysis. MESA (European and Asian ancestry) and the G6PD variant (rs1050828) in ARIC (European and African American) were not included in the discovery GWAS analysis. Effect estimates were combined in a fixed effects meta-analysis. Overall fixed effects estimates: 0.96 (0.94, 0.97), $P=3.5 \times 10^{-13}$. We note significant heterogeneity (I-squared = 89%) likely due to differences in the allele frequencies of variants used to construct ancestry-specific GS-E.

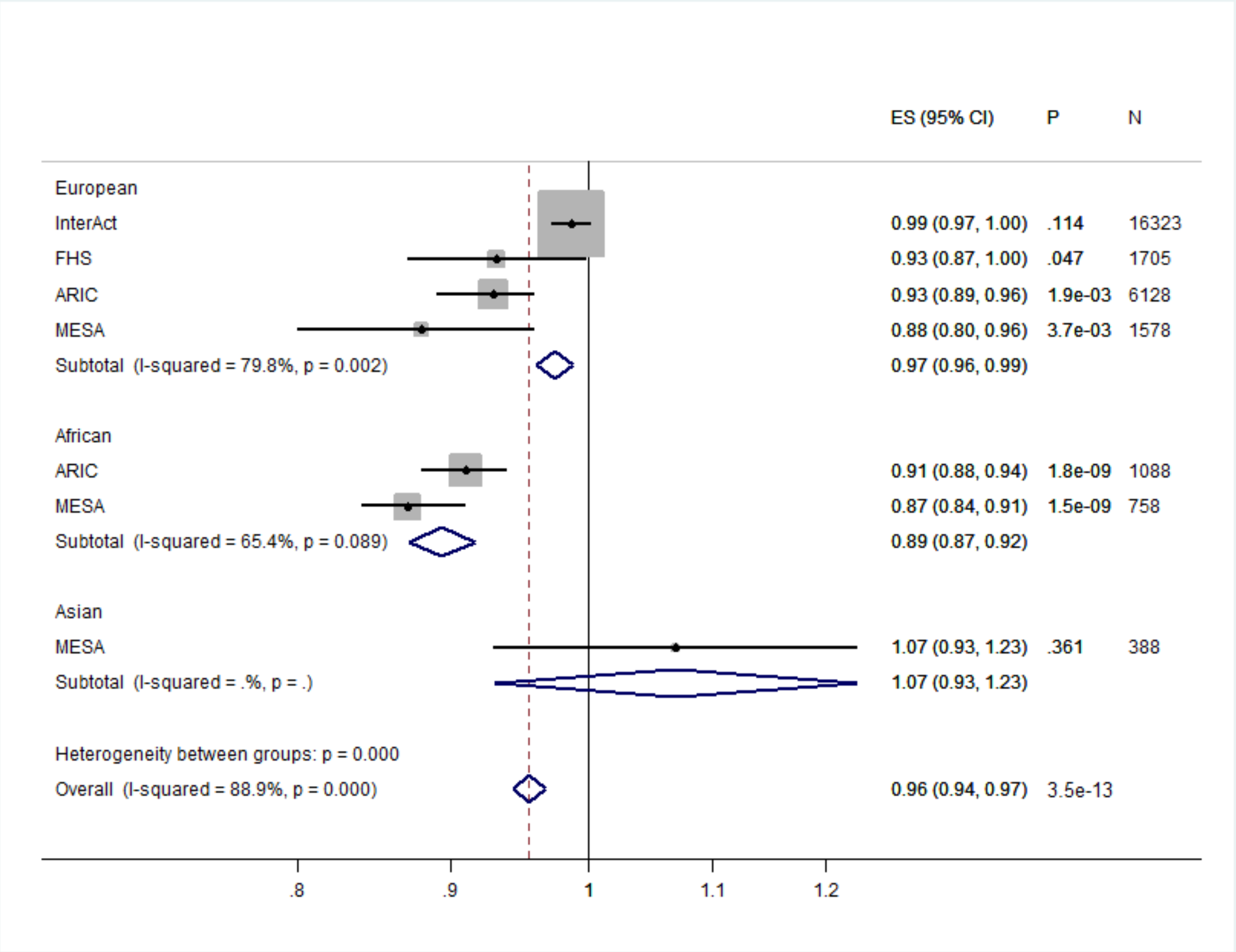

Supplement: S4 Fig — (PDF) [file pmed.1002383.s017.pdf]
