# Supplementary material for: Impact of common genetic determinants of Hemoglobin A1c on type 2 diabetes risk and diagnosis in ancestrally diverse populations: A transethnic genome-wide meta-analysis
Source: PLoS Med. 2017 Sep 12;14(9):e1002383. doi: 10.1371/journal.pmed.1002383 (PMC5595282; doi:10.1371/journal.pmed.1002383)
Supplement: S1 Analysis Plans — (DOCX) [file pmed.1002383.s018.docx]

**Overview of analysis**

1. HbA1c Genetic Discovery

Discovery meta-analysis

- Ancestry specific

- Transethnic

Primary signal identification

Identification of secondary signals using GCTA

Check all primary and secondary signals are distinct

1. Classification of all primary and secondary signals as glycemic/erythrocytic
2. Investigate the effect of glycemic and erythrocytic variants on diabetes prediction

Test the hypothesis: Glycemic and erythrocytic HbA1c-associated SNPs differentially predict incident type 2 diabetes (T2D).

1. Investigate the effect of glycemic and erythrocytic variants on diabetes classification

Test the hypothesis that erythrocytic variants may influence classification of diabetes using HbA1c?

1. Genetic Architecture of HbA1c by Ancestry

Investigate the HbA1c variance explained by all HbA1c-associated variants and how this varies by ancestry

Additional follow-up of G6PD signal

**Hemoglobin A1c Genetic Discovery Analysis Plan**

**July 2013**

**Analysis plan for cohorts joining the Hemoglobin A1c Trans-ethnic GWAS-MA effort. The analyses from each joining cohort should be performed for HbA1c.**

**Cohorts should also complete the Cohort information file separately.**

**Analysis Specifications**

Please perform and submit the analysis of **DIRECTLY GENOTYPED and IMPUTED SNPs.**

**Exclusion criteria and quality control:**

**Pre-analysis QC:**

1. Use standard exclusion criteria for sample call rate, gender checks and sample heterogeneity.
2. Use standard exclusion cut-offs for SNP call rate, HWE.
3. Include all samples and SNPs passing these criteria in analysis.
4. Align SNPs to “+” strand.
5. Other exclusions (by allele frequency, additional HWE etc.) will be made centrally.

**Additional analysis notes for GWA studies:**

Use genotyped and imputed SNPs (but only one SNP data set; where genotyped SNPs available, use them; otherwise, use imputed SNPs)

**Imputation of GWA data should be performed using HapMapII (+HapMapIII, optionally) reference panel.**

Produce a README file, whenever any additional information needs to be given about the uploaded files and QC steps (see below).

**Please, apply the following QC criteria to your data:**

1. We expect that appropriate QC criteria were applied to the genotyped SNPs in your data before the imputation was performed. We advise the following exclusions (in the advised or your own range) before you run the Genome-Wide Imputation to enable the good imputation procedure performance:

- HWE (advised p>10e-6)
- SNP call rate (advised >95%)
- MAF (advised > 1%)

Please, let us know in “Supplementary Cohort Information” file about all QC criteria applied before IMPUTATION. Cohort specific variation in the cut-offs should be recorded in the above mentioned file.

1. We advise a cut-off of proper_info>0.4 (IMPUTE users) or r2hat> 0.3 (MACH users). This will exclude poorly imputed SNPs. This QC step should be reported in the “Supplementary Cohort Information” file.
2. Exclude SNPs with MAF < 1%. This QC step should be reported in the “Supplementary Cohort Information” file.
3. Do not report SNPs twice: we advise to keep directly genotyped SNPs as they were and don’t substitute them fully with the imputed values.
4. p-values don’t need to, but can be corrected for GC (genomic control). State it clearly, if GC control was applied in the README file. This correction can be implemented during the meta-analysis, thus, is not necessary.

A**dditional QC:**

1. For case cohorts (diseases like STROKE, CAD and similar), please remove samples if
2. hospitalization or (b) blood transfusion

occurred in the 2-3 months before phenotyping took place.

**Perform analysis:**

**HbA_1C_**

1. Trait are “raw” untransformed HbA1c values in % of hemoglobin, without rank normalization
2. Exclude all individuals with Diabetes (T2D, T1D) from data sets ('diagnosed', on diabetes treatment (oral and insulin) or FPG >=7 mmol/L)
3. **If FPG is NOT available, exclude individuals with 2hr-hour glucose >= 11.1 mmol/L and/or HbA1c >= 6.5%**
4. Exclude samples with major blood abnormalities (thalassemia, sickle cell anemia, etc)
5. Exclude samples who have had a blood transfusion in the previous 2-3 months
6. Additive genetic model, 1 d.f. trend test
7. No imputation of missing traits
8. No outlier exclusion
9. Studies otherwise to apply their 'usual' adjustments (e.g. age, age2, sex, cohort, ancestry principal components, etc).
10. Analyses to be performed
    1. Without additional adjustment
    2. with BMI adjustment
    3. with Fasting glucose adjustment
11. Meta-analyses will be performed:
    1. using all European ancestry HbA1c GWAS & Metabochip data ;
    2. using all African American HbA1c GWAS data ;
    3. using all Asian GWAS & Metabochip ;
    4. pooling the Meta-analyses;
    5. we will also explore pooled meta-analysis using MANTRA (with Andrew Morris)
12. **In the cohort information file, please provide:**
    1. **The type, manufacturer and any standardization of your HbA1c assay**
    2. **Was the lab that performed the assay NGSP aligned?**
    3. **HbA1c distribution details (HbA1c mean, SD, median, min, max,** **10%, 25%, 75%, 90%iles)**

**Uploading Data and Timeline**

Please post results from above analyses to **ftp.sanger.ac.uk** using the **username 'ftp'** in the directory **pub/incoming** and email [ew2@sanger.ac.uk](mailto:ew2@sanger.ac.uk) say that they have been uploaded (please note that it is not possible to view files once they have been uploaded however they can still be retrieved from within the Sanger Inst).

**Data File General Formatting Recommendations**

- Initially, data should be reported on **NCBI BUILD 36**, and in all cases the build information should be stated in the file.

- Tab delimited text file; one row per genotyped SNP; the first row is a header with the labels given below, with the requested information in the following columns (see below).

- Filenames

CONSORTIUM_HbA1C_STUDY_DATE_INITIALS.txt.gz

CONSORTIUM _HbA1C_adjBMI_STUDY_DATE_INITIALS.txt.gz

CONSORTIUM_HbA1C_adjFG_STUDY_DATE_INITIALS.txt.gz

where:

**CONSORTIUM**: Eg. MAGIC, AAGILE, AGEN, TAICHI

**STUDY**: indicates a uniquely identifiable COHORT STUDY code (e.g. WTCCC, DGI, DGDG, FUSION, ERGO, DUNDEE, NHS, FHS, TYROL etc.; to be determined by individual participating study working groups)

**DATE**: indicates the date of file generation (DDMMMYYYY format, e.g. 17FEB2010)

**INITIALS**: indicates the initials of the individual who generated the file and/or submitted it (e.g, BFV, IP, CML, etc.)

**Specific Notes on Data Handling**

- We require use of the period character (“.”) to denote missing data or when data are not available. If a column of data are not available for a particular cohort, include the column header, and use "." to indicate missing information in each row of data.

- Generally, we acknowledge that some details of the recommended formats may not be available or may not be readily sharable (for example, BAYES_FACTOR, or N0/N1/N2 exact genotype counts). In those cases, *we recommend including* **all columns** as a general practice, but replacing the information with the missing data character (“.”).

**QTL DATA SHARING FORMAT REFERENCE TABLE (Page 1 of 1)**

| **Column header** | **Description** | **Required Format** | **Examples** |
| --- | --- | --- | --- |
| SNP | SNP label for the variant in format CHR:POS, beginning with "chr" | CHR:POS (use CHR and POS format even if rs number available) | chr1:949018 |
| rsID | rs number (if available) | rs number or Illumina/Affymetrix identifier (if no rs number available) | rs3845291 |
| STRAND | Orientation of the site to the human genome strand used | +/- acceptable, but strong preference for “+” strand orientation | + |
| BUILD | Build of the genome which the SNP is oriented on | Numeric | 36.3 |
| CHR | Chromosome on which SNP resides | Numeric for chromosomes 1-22; X for the X chromosome, Y for the Y; MT for the mitochondrial genome | 1 |
| POS | Position of SNP on chromosome | Basepairs on human genome build used | 34000345 |
| EFFECT_ALLELE | Allele at this site to which the effect has been estimated | Capital letter (A,C,G,T) | A |
| NON_EFFECT_ALLELE | Allele at this site which is not the EFFECT_ALLELE | Capital letter (A,C,G,T) | G |
| N_QTL | Total number of samples analyzed | Numeric, integer | 1243 |
| N0_QTL | Number of homozygous samples with zero copies of the EFFECT_ALLELE | Numeric, integer or float with 3 digits to the right of the decimal | 623 |
| N1_QTL | Number of heterozygous samples with one copy of the EFFECT_ALLELE | Numeric, integer or float with 3 digits to the right of the decimal | 245 |
| N2_QTL | Number of homozygous samples with two copies of the EFFECT_ALLELE | Numeric, integer or float with 3 digits to the right of the decimal | 472.268 |
| EAF_QTL | Allele frequency of the EFFECT_ALLELE | Frequency with 3 digits to the right of the decimal. Scientific E notation is acceptable. | 0.354 |
| HWE_P_QTL | Exact HWE p-value for the sample analyzed | 4 digits to the right of the decimal and use scientific E notation. | 1.0000E-02 |
| CALL_RATE_QTL | Call rate for this SNP across all subjects. Perfectly genotyped (100%) data will have a CALL_RATE = 1.000. | Frequency 3 digits to the right of the decimal. Set equal to 1.000 if IMP = 1. | 0.993 |
| BETA | Estimate of the effect size | 3 digits to the right of the decimal. Missing data is acceptable if the OR and SE are not well estimated. | 0.203 or . |
| SE | Estimated standard error on the estimate of the effect size. | 4 digits to the right of the decimal. Missing data is acceptable if the OR and SE are not well estimated. | 0.5611 or . |
| PVAL | Significance of the variant association, uncorrected for genomic control | 3 digits and use scientific E notation | 3.244E-10 |
| IMPUTED | Is the SNP imputed? | Imputed = 1  Directly-typed SNP = 0 | 1  0 |
| INFO_TYPE | Type of information provided in the INFO column | 0 = SNP is genotyped  1 = “r2_Hat” from MACH2QTL  2 = “proper_info” from SNPTEST  3 = “INFO” from PLINK | 1 |
| INFO | Measure of information content for the imputed SNP result (range 0-1) | 3 digits to the right of the decimal. Set to missing if data IMP = 0. | 0.483 |
| BAYES_FACTOR | Bayes Factor from analysis if provided by analysis program | 3 digits to the right of the decimal. Set to “.” if missing. | 4.356 or . |

**Conditional analyses in GCTA (July 2015)**

Following the discovery genome-wide meta-analysis we will run approximate conditional analysis in GCTA to identify secondary signals.

Analysis to be run by ancestry, using the ancestry-specific meta-analysis results, and individual-level genotypes as a reference sample for LD.

Attached are the lead SNPs from the transethnic MANTRA analysis split by chromosome (in the format leads_chr*.txt).

More information on conditional analyses in GCTA can be found here: <http://www.complextraitgenomics.com/software/gcta/massoc.html>

The meta-analysis results are on the sftp: /magic-incoming/GCTA/*_GCTAformat.txt

MAGIC_GCTAformat.txt (European ancestry)

AAGILE_GCTAformat.txt (African American ancestry)

EASTASIAN_GCTAformat.txt (East Asian ancestry)

SOUTHASIAN_GCTAformat.txt (South Asian ancestry)

An example command is:

./gcta64 --bfile plink_unrelated --maf 0.01 --massoc-file MAGIC_GCTAformat.txt --massoc-cond leads_chr*.txt --out chr*_MAGIC_cohort'

Please check that the *.given files contain all SNPs & upload all the output files: *.cma *.given *.badsnps & log files to the sftp folder /magic-incoming/GCTA/RESULTS.

Once all primary and secondary signals have been identified, GCTA will be run to ensure all lead variants are distinct.

**Investigate the Effect of Glycemic and Erythrocytic Hemoglobin A1c (HbA1c) Genetic Variants on Diabetes Prediction**

**June 2015**

**Background:** Mild elevations in Hemoglobin A1c (HbA1c), between 5.7 and 6.5%, generally signify “pre-diabetes”, although non-glycemic factors also contribute to HbA1c variability in the population. Previous genome-wide association studies (GWAS) have uncovered 17 HbA1c-associated loci. In an ongoing **trans-ethnic meta-analysis GWAS**, 43 novel HbA1c-associated loci have been uncovered.

**Aim:** We seek to determine the influence of glycemic, erythrocytic and unclassified HbA1c-associated SNPs on incident T2D risk in people of different ethnicities.

**Hypothesis 1:** Glycemic and erythrocytic HbA1c-associated SNPs differentially predict incident type 2 diabetes (T2D).

**Hypothesis 2:** Among non-T2D individuals with elevated HbA1c, carriers of erythrocytic HbA1c-raising alleles are likely to have dysglycemia, and progress to overt T2D. The association between erythrocytic HbA1c-raising alleles and incident T2D risk is independent of clinical variables - Fasting Glucose (FG), Body-Mass-Index (BMI) and T2D family history.

**Analysis plan for individual cohorts**

Cohorts

| **Cohort** | **Race/Ethnicity** | **Contact** |
| --- | --- | --- |
| Framingham Heart Study (FHS) | European Americans | Aaron Leong  Bianca Porneala  James Meigs |
| EPIC-InterAct | European | Stephen Sharp  Clara Podmore  Robert Scott  Claudia Langenberg |
| Atherosclerosis Risk in Communities Study (ARIC) | African Americans/  European Americans | Nisa Maruthur  Mandy Li |
| Singapore Chinese Health Study (SCHS) | Chinese Singaporeans | Xueling Sim  Mark Pereira  Myron Gross  E Shyong Tai |
| Multi-Ethnic Study of Atheroslerosis (MESA) | European Americans  African Americans  Asian Americans | Jerry Rotter  Xiuqing Guo |

Population

*Inclusion criteria*

1. Non-T2D participants at baseline

*Exclusion criteria*

Any **one** of the following:

1. FG ≥ 7 mmol/L
2. Random or 2-hour glucose ≥ 11.1 mmol/L (if available in your cohort)
3. Physician diagnosis for T2D (if available in your cohort)
4. Anti-diabetic medication use

Genetic scores (GS)

The rsID of 60 novel and known HbA1c SNPs, ethnic-specific effect alleles (HbA1c-raising alleles), ethnic-specific beta coefficients (weights), and classification (glycemic **[G]** vs. erythrocytic **[E]** vs. unclassified **[U]**) are provided in a separate attachment.

**We will generate four GS.**

1. **GS-G,** comprising HbA1c-raising alleles at up to **19** **glycemic SNPs**
2. **GS-E,** comprising HbA1c-raising alleles at up to **22 erythrocytic SNPs**
3. **GS-U,** comprising HbA1c-raising alleles at up to **19 unclassified SNPs**
4. **GS-Total,** comprising HbA1c-raising alleles at up to **60 SNPs**

*****The classification variable, GS CLASSIFICATION, is in column S**.

**Some of the SNPs had beta coefficients that were in the opposite direction to EUR, the coded allele should be “flipped” so that all ASN and AFR HbA1c-raising alleles become effect alleles.**

**SNPs in LD (R2>0.8) can be used as proxies for missing index SNPs.**

*Weights*

We will apply weights derived from ethnic-specific beta coefficients **(column V, Y or AB)** using the following formula:

weighted GS = *n**(beta1*SNP1 + beta2*SNP2 +…..beta*j**SNP*j*) / (beta1 + beta2 +….beta*j*)

where  *j* indexes SNPs

*n* = total number of SNPs in the GS

beta = point estimate for the beta coefficient of the SNP

SNP = number of HbA1c-raising allele i.e. 0, 1 or 2

e.g. An individual’s SNP profile is as follows: SNP1 = 2, SNP2 = 1, SNP3 = 0, SNP4 = 2; beta1 = 0.01, beta2 = 0.02, beta3 = 0.03, beta4 = 0.04

unweighted GS = 2 + 1 + 0 + 2 = 5

weighted GS = 4 x (0.01 x 2 + 0.02 x 1 + 0.03 x 0 + 0.04 x 2) / (0.01 + 0.02 + 0.03 + 0.04) = 4.8

Outcome

**Incident T2D** over the follow-up period (≥ 10 years), defined as any **one** of the following:

1. Physician diagnosis for T2D (if available in your cohort)
2. Anti-diabetic medication use
3. FG ≥ 7mmol/L

**Note: We will not use HbA1c to define the outcome (incident T2D).**

Usual covariates for adjustment in **all models**

1. Sex
2. Age
3. Study design variables (i.e. clinic sites, recruitment cohort)
4. Principal Components (PCs) to account for population substructure (if appropriate for your study)

Clinical covariates for adjustment in **some of the models**

1. FG/glucose (continuous variable)
2. BMI (continuous variable)
3. Family history for T2D (binary variable: yes vs. no)
4. HbA1c (**binary variable**: <5.7% vs. ≥5.7%; this variable is used in the interaction and clinical models)

Statistical analysis

1. *GS effect on baseline HbA1c levels – linear regression*

We will run the following models for baseline HbA1c **as a continuous variable**, adjusted for usual covariates:

| **Models** | **Variables** |
| --- | --- |
| **Genetic** | **HbA1c (continuous variable) ~ GS-G** |
| **Genetic** | **HbA1c (continuous variable) ~ GS-E** |
| **Genetic** | **HbA1c (continuous variable) ~ GS-U** |

1. *GS effect on incident T2D - logistic regression/Cox regression*

In the entire cohort, we will run the following models, adjusted for usual covariates:

| **Models** | **Variables** |
| --- | --- |
| **Genetic** | **T2D ~ GS-G + GS-E + GS-U** |
| **Glycemic** | **T2D ~ GS-G + GS-E + GS-U + glucose** |
| **HbA1c-adjusted (1)** | **T2D ~ GS-E + HbA1c (binary variable)** |
| **HbA1c-adjusted (2)** | **T2D ~ GS-E + HbA1c (continuous variable)** |

Data sharing

**Please report results in the following tables:**

1. *Baseline characteristics.* Please provide **mean/median/standard deviation** for continuous variables and **n(%)** for categorical variables

| **Entire cohort** | **No T2D during follow-up** | **Incident T2D during follow-up** |
| --- | --- | --- |
| N |  |  |
| Age (years) |  |  |
| Female; n |  |  |
| FG/glucose (mmol/L) |  |  |
| HbA1c (% points) |  |  |
| BMI (kg/m^2^) |  |  |
| Family history |  |  |
| GS-G |  |  |
| GS-E |  |  |
| GS-U |  |  |

1. *Effect of weighted HbA1c-raising allele on baseline HbA1c levels (% points)*

Variance explained is by the GS only, i.e., the difference in the variance explained by the “full” model (e.g. GS-G, age and sex) and the “reduced model” with only the usual covariates (age and sex).

| **GS** | **Beta** | **SE** | **95%CI** | **P** | **N** | **Variance explained** |
| --- | --- | --- | --- | --- | --- | --- |
| **GS-G** |  |  |  |  |  |  |
| **GS-E** |  |  |  |  |  |  |
| **GS-U** |  |  |  |  |  |  |

1. *Effect of GS on incident T2D*

|  | **Models** | **Variable** | **HR/OR** | **SE** | **95LL** | **95UL** | **P** | **N** |
| --- | --- | --- | --- | --- | --- | --- | --- | --- |
| Entire cohort | T2D ~ GS-G + GS-E + GS-U | GS-G |  |  |  |  |  |  |
|  |  | GS-U |  |  |  |  |  |  |
|  |  | GS-E |  |  |  |  |  |  |
|  | T2D ~ GS-G + GS-E + GS-U + glucose | GS-G |  |  |  |  |  |  |
|  |  | GS-U |  |  |  |  |  |  |
|  |  | GS-E |  |  |  |  |  |  |
|  | T2D ~ GS-G | GS-G |  |  |  |  |  |  |
|  | T2D ~ GS-G + glucose | GS-G |  |  |  |  |  |  |
|  | T2D ~ GS-U | GS-U |  |  |  |  |  |  |
|  | T2D ~ GS-U + glucose | GS-U |  |  |  |  |  |  |
|  | T2D ~ GS-E | GS-E |  |  |  |  |  |  |
|  | T2D ~ GS-E + glucose | GS-E |  |  |  |  |  |  |
|  | T2D ~ GS-E + HbA1c (binary) | GS-E |  |  |  |  |  |  |
|  | T2D ~ GS-E _ HbA1c(continuous) | GS-E |  |  |  |  |  |  |

**Net-reclassification analysis (estimating the effect of the erythrocytic loci on T2D classification) & total explained variance**

**Sep 2015**

**Net-reclassification**

1. Exclude physician-diagnosed diabetes/on diabetes medication, leaving a sample of people without treated/clinical diabetes that can be screened to detect undiagnosed diabetes. Divide

the rest by FG <126 mg/dl (no T2D) or =>126 mg/dl (7 mmol/l)(undiagnosed T2D).

2. Show the distribution of numbers of people in HbA1c bins of 0.1(%) from <5.8% to >=6.7%. This is the crude population distribution by HbA1c (see table below).

3. Estimate the level of HbA1c, adjusted for the ~20 erythrocytic SNPs (list of SNPs will be provided in separate attachment).

4. Take the SNP-adjusted HbA1c levels and re-distribute people by HbA1c level as above, by 0.1(%) bins (information below).

5. The table below shows the data display required, for 0.1(%) bins.

**6. Repeat 1 through 5, now dividing the population by FG<100 mg/dl (no T2D), or >= 100 (5.56 mmol/l) (impaired fasting glucose/undiagnosed T2D), and show the distribution of numbers of people in HbA1c bins of 0.1(%) from <4.8% to >=6.7%.**

6. Centrally, we will add the numbers of people in each of the crude and adjusted cells across the studies, and from this summary distribution estimate the net reclassification improvement.

7. Report the mean, median, standard deviation, maximum and minimum value for (a) unadjusted HbA1c, (b) SNP-adjusted HbA1c and (c) the difference between the SNP-adjusted HbA1c and unadjusted HbA1c.

**Example table to be completed by all cohorts:**

Justification:

We want to adjust hba1c for known SNPs, but we want the resulting distribution to be similar to the original distribution (at least have the same mean). The residuals from a regression on the ~20 SNPs will produce “adjusted” values, but residuals will have mean of 0. So, the proper adjusted hba1c values to use are:

$$\bar{Y}+r_{i}$$

Where $\bar{Y}$ is the cohort specific mean hba1c and *r_i_* is the residual from the regression for individual i

I will show you that what I am proposing is equivalent to the above.

$$r_{i}=Y_{i}-\hat{Y}_{i}$$

Let *g_ki_* is the dosage genotype for the k^th^ SNP for individual i and let $\hat{\beta}_{k}$be the SNP effects (estimated from the replication meta-analysis). Remember that the intercept estimate for the regression equation adjusting for the 7 SNPs can be written as

Then

Note that E(g_ki_) is just the average dosage or 2 × effect allele frequency.

The adjusted value we want to use can be written as:

**Example calculation of adjusted HbA1c:**

gen adja1c3= rcal_a1c - 0.0256*((2- rs2779116)-(2*(1-0.7362))) - 0.0588*(rs1800562-2*0.9361) - 0.0255*( rs4737009-2*0.2386) - 0.0903*( rs16926246-2*0.9096) - 0.0277*((2-rs7998202)-(2*(1-0.8427))) - 0.0357*((2- rs1046896)-(2*(1-0.6854))) - 0.0259*((2- rs855791)-(2*(1-0.5612)))

**HbA1c Variance Explained by HbA1c-associated Variants**

To estimate the total explained variance of HbA1c, calculate a regression model including all genome-wide significant SNPs (see Appendix). Please use the complete set of individuals used for the primary meta-analysis. If the individual genotypes of the SNPs are called geno1, geno2, etc., then fit a regression model

HbA1c = intercept + b1*geno1 + b2*geno2 + ... + sex + age + usual covariates + error

and report the estimates, standard errors and p-values, for the coefficients b1, b2, etc. This can be done in any standard statistical software. Finally please report the total explained variance by the genome-wide significant SNPs used in the analysis and the sample size of this joint analysis. This should be done by comparing the explained variances of the full model with all SNPs and a basic model including only sex, age and the other covariates. How you will get these numbers depends on your statistical software. If you have problems we are happy to assist you in doing these calculations.

ADDENDUM:

1. calculate the variance explained by the ~20 erythrocytic HbA1c SNPs
2. calculate the variance explained by the ~60 HbA1c SNPs

**The Difference Between Fructosamine-inferred HbA1c and Measured HbA1c**

**Nov 2015**

HbA1c ~ fructosamine

-> obtain residuals

Then

residuals ~ rs1050828 + age + sex + usual covariates

**Report the following:**

1. mean, SD, median, min, max for the residuals of HbA1c ~ fructosamine, by genotype (C, T, CC, CT, TT)

| **Genotype** | **Variable** | **Mean** | **SD** | **median** | **min** | **max** | **N** |
| --- | --- | --- | --- | --- | --- | --- | --- |
| **Men C** | **Res** |  |  |  |  |  |  |
| **Men T** | **Res** |  |  |  |  |  |  |
| **Women CC** | **Res** |  |  |  |  |  |  |
| **Women CT** | **Res** |  |  |  |  |  |  |
| **Women TT** | **Res** |  |  |  |  |  |  |

1. mean, SD, median, min, max for fructosamine by genotype (C, T, CC, CT, TT)

what is the units for fructosamine? ___________________

| **Genotype** | **Variable** | **Mean** | **SD** | **median** | **min** | **Max** | **N** |
| --- | --- | --- | --- | --- | --- | --- | --- |
| **Men C** | **Fructosamine** |  |  |  |  |  |  |
| **Men T** | **Fructosamine** |  |  |  |  |  |  |
| **Women CC** | **Fructosamine** |  |  |  |  |  |  |
| **Women CT** | **Fructosamine** |  |  |  |  |  |  |
| **Women TT** | **Frucotsamine** |  |  |  |  |  |  |

1. Report the following separately for AA, AA(F), AA(M)

|  | **Analysis** | **Exposure** | **Outcome** | **Beta** | **SE** | **95LL** | **95UL** | **P** | **N** |
| --- | --- | --- | --- | --- | --- | --- | --- | --- | --- |
| **All AA** | **4** | rs1050828 | **Res** |  |  |  |  |  |  |
| **Men AA** | **4** | rs1050828 | **Res** |  |  |  |  |  |  |
| **Women AA** | **4** | rs1050828 | **Res** |  |  |  |  |  |  |

**“Exclude anemia” analysis plan**

**EXCLUDE ALL PARTICIPANTS ON DIABETES MEDICATION OR WITH PHYSICIAN-DIAGNOSED DIABETES**

**In AA,**

1. HbA1c ~ rs1050828 + age + sex + usual covariates
2. HbA1c ~ rs1050828 + age + sex + Hb + usual covariates

**In AA women with Hb>=12 g/dL,**

1. HbA1c ~ rs1050828+ age + usual covariates

**In AA men with Hb>=13g/dL.**

1. HbA1c ~ rs1050828 + age + usual covariates

**In AA men with Hb >=13g/dL and AA women with Hb >= 12 g/dL and one group**

1. HbA1c ~ SNP + age + sex + usual covariates

| **Analysis** | **Exposure** | **Outcome** | **Beta** | **SE** | **95LL** | **95UL** | **P** | **N** |
| --- | --- | --- | --- | --- | --- | --- | --- | --- |
| **1** | rs1050828 | **HbA1c** |  |  |  |  |  |  |
| **2** | rs1050828 | **HbA1c** |  |  |  |  |  |  |
| **3** | rs1050828 | **HbA1c** |  |  |  |  |  |  |
| **4** | rs1050828 | **HbA1c** |  |  |  |  |  |  |
| **5** | rs1050828 | **HbA1c** |  |  |  |  |  |  |
