# Supplementary material for: Impact of common genetic determinants of Hemoglobin A1c on type 2 diabetes risk and diagnosis in ancestrally diverse populations: A transethnic genome-wide meta-analysis
Source: PLoS Med. 2017 Sep 12;14(9):e1002383. doi: 10.1371/journal.pmed.1002383 (PMC5595282; doi:10.1371/journal.pmed.1002383)
Supplement: S1 Financial Disclosure — (DOCX) [file pmed.1002383.s019.docx]

**The funders had no role in study design, data collection and analysis, decision to publish, or preparation of the manuscript.**

(AASC) This work was supported by Grants for Scientific Research (24390084, 21390099, 20390185) from the Ministry of Education, Culture, Sports, Science, and Technology, Japan; a Science and Technology Incubation Program in Advanced Regions, Japan Science and Technology Agency; the Japan Atherosclerosis Prevention Fund; the Takeda Medical Research Foundation; and National Cardiovascular Research Grants.

(AMC_PAS) The department of vascular medicine provided the resources for the conduct of this study. GKH is funded by a VIDI grant provided by the Dutch Science Organisation (no. 016.156.445) and supported by CardioVascular Research Initiative [CVON2011-19; Genius] and the European Union [TransCard: FP7-603091-2]

Andrew P Morris is a Wellcome Trust Senior Fellow in Basic Biomedical Science (grant number WT098017)

(ARIC) The Atherosclerosis Risk in Communities Study is carried out as a collaborative study supported by National Heart, Lung, and Blood Institute contracts (HHSN268201100005C, HHSN268201100006C, HHSN268201100007C, HHSN268201100008C, HHSN268201100009C, HHSN268201100010C, HHSN268201100011C, and HHSN268201100012C), R01HL087641, R01HL59367 and R01HL086694; National Human Genome Research Institute contract U01HG004402; and National Institutes of Health contract HHSN268200625226C. Infrastructure was partly supported by Grant Number UL1RR025005, a component of the National Institutes of Health and NIH Roadmap for Medical Research. Dr. Selvin is supported by 2R01DK089174 and K24DK106414. Reagents for the fructosamine assays were donated by Roche Diagnostics. Funding for the HbA1c measurements in ARIC was originally provided by R21DK080294 to Dr. Selvin. Dr. Li received support from a National Heart, Lung, and Blood Institute T32-HL007024 Cardiovascular Epidemiology Training Grant.

(B58C-T1DGC) This research utilizes resources provided by the Type 1 Diabetes Genetics Consortium, a collaborative clinical study sponsored by the National Institute of Diabetes and Digestive and Kidney Diseases (NIDDK), National Institute of Allergy and Infectious Diseases (NIAID), National Human Genome Research Institute (NHGRI), National Institute of Child Health and Human Development (NICHD), and Juvenile Diabetes Research Foundation International (JDRF) and supported by U01 DK062418. T1DGC GWAS data were deposited by the Diabetes and Inflammation Laboratory, Cambridge Institute for Medical Research (CIMR), University of Cambridge (John Todd, Helen Stevens and Neil Walker), which is funded by Juvenile Diabetes Research Foundation International, the Wellcome Trust and the National Institute for Health Research Cambridge Biomedical Research Centre; the CIMR is in receipt of a Wellcome Trust Strategic Award (079895).

(B58C-WTCCC) We acknowledge use of genotype data from the British 1958 Birth Cohort DNA collection, funded by the Medical Research Council grant G0000934 and the Wellcome Trust grant 068545/Z/02. Genotyping for the Wellcome Trust Case Control Consortium was funded by the Wellcome Trust grant 076113/B/04/Z.

Bengt Sennblad acknowledges funding from the Magnus Bergvall foundation and the Foundation for old servants

(BioMe) The Mount Sinai IPM BioMe Program is supported by The Andrea and Charles Bronfman Philanthropies.

(BLSA) The BLSA was supported by the Intramural Research Program of the NIH, National Institute on Aging.

(CAGE) The CAGE Network studies were supported by grants for the Core Research for Evolutional Science and Technology (CREST) from the Japan Science Technology Agency; the Program for Promotion of Fundamental Studies in Health Sciences, National Institute of Biomedical Innovation Organization (NIBIO); and the Grant of National Center for Global Health and Medicine (NCGM).

(CARDIA) The Coronary Artery Risk Development in Young Adults Study (CARDIA) is funded by the National Heart, Lung, and Blood Institute in collaboration with the University of Alabama at Birmingham (HHSN268201300025C & HHSN268201300026C), Northwestern University (HHSN268201300027C), University of Minnesota (HHSN268201300028C), Kaiser Foundation Research Institute (HHSN268201300029C), and Johns Hopkins University School of Medicine (HHSN268200900041C).  CARDIA is also partially supported by the Intramural Research Program of the National Institute on Aging. Genotyping of the CARDIA participants was supported by grants U01-HG-004729, U01-HG-004446, and U01-HG-004424 from the National Human Genome Research Institute.

Clara Podmore is funded by the Wellcome Trust (grant 097451/Z/11/Z)

(CROATIA) CROATIA Korcula and Vis is supported by the Medical Research Council UK, the Croatian Ministry of Science, Education and sports (grant 216-1080315-0302) and Croatian Science Foundation (grant 8875).

(D.E.S.I.R) The D.E.S.I.R. study has been supported by INSERM contracts with CNAMTS, Lilly, Novartis Pharma and Sanofi-Aventis; by INSERM (Réseaux en Santé Publique, Interactions entre les déterminants de la santé), Cohortes Santé TGIR, the Association Diabète Risque Vasculaire, the Fédération Française de Cardiologie, LA Fondation de France, ALFEDIAM, Société francophone du diabète, ONIVINS, Abbott, Ardix Medical, Bayer Diagnostics, Becton Dickinson, Cardionics, Merck Santé, Novo Nordisk, Pierre Fabre, Roche, Topcon.

David J. Roberts is funded by NHS Blood and Transplant (UK) and the NIHR Biomedical Research Centre Programme at Oxford and the National Institute for Health Research Blood and Transplant Research Unit (NIHR BTRU) in Donor Health and Genomics.

(DIAGEN) The DIAGEN study was supported by the Commission of the European Communities, Directorate C - Public Health and Risk Assessment, Health & Consumer Protection, Grant Agreement number - 2004310 and by the Dresden University of Technology Funding Grant, Med Drive.

(DPS) The DPS has been financially supported by grants from the Academy of Finland (117844 and 40758, 211497, and 118590 (MU); The EVO funding of the Kuopio University Hospital from Ministry of Health and Social Affairs (5254), Finnish Funding Agency for Technology and Innovation (40058/07), Nordic Centre of Excellence on ‘Systems biology in controlled dietary interventions and cohort studies, SYSDIET (070014), The Finnish Diabetes Research Foundation, Yrjö Jahnsson Foundation (56358), Sigrid Juselius Foundation and TEKES grants 70103/06 and 40058/07.

Erik Ingelsson acknowledges funding from NIH grant 1R01DK106236-01A1

(FHS/MGH/BU) This research was conducted in part using data and resources from the Framingham Heart Study of the National Heart Lung and Blood Institute of the National Institutes of Health and Boston University School of Medicine. This work was partially supported by the National Heart, Lung and Blood Institute's Framingham Heart Study (contract number N01‐HC‐25195) and its contract with Affymetrix, Inc for genotyping services (contract number N02‐HL‐6‐4278). The work is also supported by National Institute for Diabetes and Digestive and Kidney Diseases (NIDDK) R01 DK078616 to D.V.R, J.H, J.B.M., J.D., C.-T.L. and J.C.F., NIDDK K24 DK080140 to J.B.M., NIDDK U01 DK085526 to J.D., J.B.M. and J.C.F., a MGH Research Scholars Award to J.C.F, a Canadian Diabetes Association Postdoctoral Research fellowship to A.L., and an American Diabetes Association Pathway Accelerator Award #1-15-ACE-26 to M.F.H.

(FIN-D2D 2007) The FIN-D2D 2007 study was supported by funds from the hospital districts of Pirkanmaa; Southern Ostrobothnia; North Ostrobothnia; Central Finland and Northern Savo; the Finnish National Public Health Institute; the Finnish Diabetes Association; the Ministry of Social Affairs and Health in Finland; Finland’s Slottery Machine Association; the Academy of Finland [grant number 129293] and Commission of the European Communities, Directorate C-Public Health [grant agreement no. 2004310].

(GoDARTS) The UK Type 2 Diabetes Genetics Consortium collection was supported by the Wellcome Trust (Biomedical Collections Grant GR072960).

(HANDLS) The Healthy Aging in Neighborhoods of Diversity across the Life Span (HANDLS) study was supported by the Intramural Research Program of the NIH, National Institute on Aging and the National Center on Minority Health and Health Disparities (project # Z01-AG000513 and human subjects protocol number 09-AG-N248).

(Health ABC) This research was supported by NIA contracts N01AG62101, N01AG62103, and N01AG62106. The genome-wide association study was funded by NIA grant 1R01AG032098-01A1 to Wake Forest University Health Sciences and genotyping services were provided by the Center for Inherited Disease Research (CIDR). CIDR is fully funded through a federal contract from the National Institutes of Health to The Johns Hopkins University, contract number HHSN268200782096C. This research was supported in part by the Intramural Research Program of the NIH, National Institute on Aging.

(InterAct) Funding for the InterAct project was provided by the EU FP6 programme (grant number LSHM_CT_2006_037197). In addition, InterAct investigators acknowledge funding from the following agencies: PD: Wellcome Trust; MIM: Wellcome Trust (083270/Z/07/Z), MRC (G0601261); PWF: Swedish Research Council, Novo Nordisk, Swedish Diabetes Association, Swedish Heart-Lung Foundation; RK: German Cancer Aid, German Ministry of Research (BMBF); TJK: Cancer Research UK C8221/A19170 and Medical Research Council MR/M012190/1; CN: Health Research Fund (FIS) of the Spanish Ministry of Health; Murcia Regional Government (Nº 6236); PMN: Swedish Research Council; KO and AT: Danish Cancer Society; SP: Compagnia di San Paolo; JRQ: Asturias Regional Government; OR: The Västerboten County Council; AMWS and YTvdS: EPIC Bilthoven and Utrecht acknowledge the Dutch Ministry of Public Health, Welfare and Sports (VWS), Netherlands Cancer Registry (NKR), LK Research Funds, Dutch Prevention Funds, Dutch ZON (Zorg Onderzoek Nederland), World Cancer Research Fund (WCRF), Statistics Netherlands; RT: Sicilian Regional Government and AIRE -ONLUS Ragusa; ER: Imperial College Biomedical Research Centre.

Inês Barroso acknowledges funding from Wellcome Trust grant WT098051

Iva Miljkovic is funded (but not for the research presented in this manuscript) by the grant from the National Institute of Health R01-DK097084.

(Jackson Heart Study) The JHS is supported by contracts HHSN268201300046C, HHSN268201300047C, HHSN268201300048C, HHSN268201300049C, HHSN268201300050C from the National Heart, Lung, and Blood Institute and the National Institute on Minority Health and Health Disparities.

Jana V. Van Vliet-Ostaptchouk is supported by a Diabetes Funds Junior Fellowship from the Dutch Diabetes Research Foundation (project no. 2013.81.1673).

(KARE) This work was supported by grants from Korea Centers for Disease Control and Prevention (4845–301, 4851–302, 4851–307), and an intramural grant from the Korea National Institute of Health (2014-NI73001-00).

(KORA) The KORA study was initiated and financed by the Helmholtz Zentrum München – German Research Center for Environmental Health, which is funded by the German Federal Ministry of Education and Research (BMBF) and by the State of Bavaria. Furthermore, KORA research was financed by a grant from the BMBF to the German Center for Diabetes Research (DZD) and a grant from the Ministry of Innovation, Science, Research and Technology of the state North Rhine-Westphalia (Düsseldorf, Germany). It was also supported within the Munich Center of Health Sciences (MC-Health), Ludwig-Maximilians-Universität, as part of LMUinnovativ.

(Leipzig_adults) This work was supported by the Kompetenznetz Adipositas (Competence network for Obesity) funded by the Federal Ministry of Education and Research (German Obesity Biomaterial Bank; FKZ 01GI1128).This work was supported by the Federal Ministry of Education and Research (BMBF), Germany, FKZ: 01EO1501 (K6e/2 to Y.B., AD2-060E to P.K.). This project was further supported by grants from the Collaborative Research Center funded by the German Research Foundation (CRC 1052; B01, B03), from the German Diabetes Association and from the DHFD (Diabetes Hilfs- und Forschungsfonds Deutschland) and from Boehringer Ingelheim Foundation.

(Leipzig-kid) The Leipzig Childhood Obesity cohort is supported by grants from Integrated Research and Treatment Centre (IFB) Adiposity Diseases FKZ: 01EO1001, from the German Research Foundation for the Clinical Research Center “Obesity Mechanisms” CRC1052/1 C05 and the European Community’s Seventh Framework Programme (FP7/2007-2013) project Beta-JUDO under grant agreement n° 279153. LIFE – Leipzig Research Center for Civilization Diseases, University Leipzig. LIFE is funded by means of the European Union, by the European Regional Development Fund (ERDF) and by means of the Free State of Saxony within the framework of the excellence initiative.

(Lifelines) The Lifelines Cohort Study, and generation and management of GWAS genotype data for the Lifelines Cohort Study is supported by the Netherlands Organization of Scientific Research NWO (grant 175.010.2007.006), the Economic Structure Enhancing Fund (FES) of the Dutch government, the Ministry of Economic Affairs, the Ministry of Education, Culture and Science, the Ministry for Health, Welfare and Sports, the Northern Netherlands Collaboration of Provinces (SNN), the Province of Groningen, University Medical Center Groningen, the University of Groningen, Dutch Kidney Foundation and Dutch Diabetes Research Foundation.

(LOLIPOP) The LOLIPOP study is supported by the National Institute for Health Research (NIHR) Comprehensive Biomedical Research Centre Imperial College Healthcare NHS Trust, the British Heart Foundation (SP/04/002), the Medical Research Council (G0601966, G0700931), the Wellcome Trust (084723/Z/08/Z), the NIHR (RP-PG-0407-10371), European Union FP7 (EpiMigrant, 279143) and Action on Hearing Loss (G51). We thank the National Institute for Health Research (NIHR) Comprehensive Biomedical Research Centre Imperial College Healthcare NHS Trust for supporting our work. The work was carried out in part at the NIHR/Wellcome Trust Imperial Clinical Research Facility.

(LURIC) LURIC was supported by the 7th Framework Program (integrated project AtheroRemo, grant agreement number 201668 and RiskyCAD, grant agreement number 305739) of the European Union and by the INTERREG IV Oberrhein Program (Project A28, Genetic mechanisms of cardiovascular diseases) with support from the European Regional Development Fund (ERDF) and the Wissenschaftsoffensive TMO.

Mike A. Nalls’ participation is supported by a consulting contract between Data Tecnica International and the National Institute on Aging, NIH, Bethesda, MD, USA

Mark I. McCarthy is a Wellcome Trust Senior Investigator (Wellcome Trust grant numbers 090532, 098381)

(MESA) MESA and the MESA SHARe project are conducted and supported by the National Heart, Lung, and Blood Institute (NHLBI) in collaboration with MESA investigators. Support for MESA is provided by contracts N01-HC-95159, N01-HC-95160, N01-HC-95161, N01-HC-95162, N01-HC-95163, N01-HC-95164, N01-HC-95165, N01-HC-95166, N01-HC-95167, N01-HC-95168, N01-HC-95169, UL1-TR-001079, UL1-TR-000040, and DK063491.

(METSIM) The METSIM study was supported by the Academy of Finland (contract 124243), the Finnish Heart Foundation, the Finnish Diabetes Foundation, Tekes (contract 1510/31/06), and the Commission of the European Community (HEALTH-F2-2007 201681).

Mika Kivimaki is supported by the UK Medical Research Council (K013351).

(NHAPC) The NHPAC study is supported by the Major Project of the Ministry of Science and Technology of China (2016YFC1304903), and the National Natural Science Foundation of China (30930081, 81471013, and 81170734).

Nicole Soranzo's research is supported by the Wellcome Trust (Grant Codes WT098051 and WT091310), the EU FP7 (EPIGENESYS Grant Code 257082 and BLUEPRINT Grant Code HEALTH-F5-2011-282510) and the National Institute for Health Research Blood and Transplant Research Unit (NIHR BTRU) in Donor Health and Genomics at the University of Cambridge in partnership with NHS Blood and Transplant (NHSBT).

(NSHD) This work was funded by the Medical Research Council (MC_UU_12019/1), the British Heart Foundation (RG/10/12/28456) and the Wellcome Trust (088869/B/09/Z).

(NTR) Funding was obtained from the Netherlands Organization for Scientific Research (NWO: MagW/ZonMW grants 904-61-090, 985-10-002, 904-61-193,480-04-004, 400-05-717, Addiction-31160008 Middelgroot-911-09-032, Spinozapremie 56-464-14192), Center for Medical Systems Biology (CSMB, NWO Genomics), NBIC/BioAssist/RK(2008.024), Biobanking and Biomolecular Resources Research Infrastructure (BBMRI –NL, 184.021.007), the VU University’s Institute for Health and Care Research (EMGO+ ) and Neuroscience Campus Amsterdam (NCA), the European Science Foundation (ESF, EU/QLRT-2001-01254), the European Community's Seventh Framework Program (FP7/2007-2013), ENGAGE (HEALTH-F4-2007-201413); the European Science Council (ERC Advanced, 230374), Rutgers University Cell and DNA Repository (NIMH U24 MH068457-06), the Avera Institute, Sioux Falls, South Dakota (USA) and the National Institutes of Health (NIH, R01D0042157-01A). Part of the genotyping and analyses were funded by the Genetic Association Information Network (GAIN) of the Foundation for the US National Institutes of Health, the (NIMH, MH081802).

(ORCADES) ORCADES was supported by the Chief Scientist Office of the Scottish Government (CZB/4/276 and CZB/4/710), the Royal Society, the MRC Human Genetics Unit, Arthritis Research UK and the European Union framework program 6 EUROSPAN project (contract no. LSHG-CT-2006-018947). DNA extractions were performed at the Wellcome Trust Clinical Research Facility in Edinburgh.

(PROCARDIS) The EU FP7 Program (LSHM‐CT‐ 2007‐037273), AstraZeneca, the British Heart Foundation, the Oxford BHF Centre of Research Excellence, the Wellcome Trust (075491/Z/04), the Swedish Research Council, the Knut and Alice Wallenberg Foundation, the Swedish Heart‐Lung Foundation, the Torsten and Ragnar Söderberg Foundation, the Strategic Cardiovascular Program of Karolinska Institutet and Stockholm County Council, the Foundation for Strategic Research and the Stockholm County Council (560283)

(PROMIS) Genotyping in PROMIS was funded by the Wellcome Trust. Fieldwork in the PROMIS study has been supported through funds available to investigators at the Center for Non-Communicable Diseases, Pakistan and the University of Cambridge, UK.

(SardiNIA) This work was supported by the Intramural Research Program of the National Institute on Aging(NO1-AG-1–2109, 263-MA-410953), and by the National Institutes of Health (HG002651, HG005581, HL084729). The funders had no role in study design, data collection and analysis, decision to publish, or preparation of the manuscript.

(SCHS-CHD - cases and controls) The funding for genotyping of this cohort was supported by the HUJ-CREATE Programme of the National Research Foundation, Singapore (Project Number 370062002). The Singapore Chinese Health Study was supported by the U.S. National Institutes of Health Grant Numbers RO1CA55069, R35CA53890, R01CA80205 and R01CA144034 and UM1 CA182876, and by the Singapore National Medical Research Council Grant Number 1270/2010.

(Segovia) This work was supported by grants FEDER 2FD 1997/2309 from the Fondo Europeo para el Desarrollo Regional, Red de Centros RCMN (C03/08), FIS 03/1618, from Instituto de Salud Carlos III-RETIC RD06/0015/0012, Madrid, Spain. We also acknowledge CIBER in Diabetes and Associated Metabolic Disorders (ISCIII, Ministerio de Ciencia e Innovación) and Madrid Autonomous Community (MOIR S2010/BMD-2423). Partial support also came from Educational Grants from Eli Lilly Lab, Spain, Bayer Pharmaceutical Co., Spain and Fundación Mutua Madrileña 2008, Spain.

(SHIP) SHIP is part of the Community Medicine Research net of the University Medicine of Greifswald, Germany, which is funded by the Federal Ministry of Education and Research (grants 01ZZ9603, 01ZZ0103 and 01ZZ0403), the Ministry of Cultural Affairs as well as the Social Ministry of the Federal State of Mecklenburg - West Pomerania. Generation of genomewide data has been supported by Grants from the German Federal Ministry of Education and Research for "Zentren für Innovationskompetenz" (ZIK; BMBF grant 03ZIK012) and a joint grant from Siemens Healthcare, Erlangen, Germany and the Federal State of Mecklenburg- West Pomerania. The University of Greifswald is a member of the “Center of Knowledge Interchange“ program of the Siemens AG.

(SIGNET) The Sea Islands Genetics Network (SIGNET) was supported by R01 DK084350. The Sea Islands Genetics Network (SIGNET) was supported by R01 DK084350 (MM Sale), and consists of data from the REasons for Geographic And Racial Differences in Stroke (REGARDS) cohort, (U01 NS041588; G Howard), Project SuGAR (Sea Islands Genetic African American Registry) (W.M. Keck Foundation; WT Garvey), a South Carolina Center of Biomedical Research Excellence (COBRE) in Oral Health Project P20 RR017696 (PI: Kirkwood; Sub-award: JK Fernandes), and the Systemic Lupus Erythematosus in Gullah Health (SLEIGH) study (PI: P60 AR049459, GS Gilkeson; K23 AR052364, DL Kamen; UL1 RR029882, KT Brady).

(Sorbs) This work was supported by grants from the German Research Council (DFG - SFB 1052 “Obesity mechanisms”; A01, C01 and SPP 1629 TO 718/2-1), from the German Diabetes Association and from the DHFD (Diabetes Hilfs- und Forschungsfonds Deutschland).

(TAICHI) This study was supported by the National Health Research Institutes, Taiwan (PH-100-SP-01, BS-094-PP-01, PH-100-PP-03), the National Science Council, Taiwan (Grant Nos NSC 98-2314-B-075A-002-MY3, NSC 96-2314-B-002-151, NSC 96-2314-B-002-152, NSC 98-2314-B-002-122-MY2, NSC 100-2314-B-002-115, NSC 101-2325-002-078, 101-2314-B-075A-006-MY3), the National Taiwan University Hospital, Taiwan (NTUH 98-N1266, NTUH 100-N1775, NTUH 101-N2010, NTUH 101-N, VN101-04, NTUH 101-S1784).

(TRAILS) TRAILS (TRacking Adolescents’ Individual Lives Survey) is a collaborative project involving various departments of the University Medical Center and University of Groningen, the Erasmus University Medical Center Rotterdam, the University of Utrecht, the Radboud Medical Center Nijmegen, and the Parnassia Bavo group, all in the Netherlands. TRAILS has been financially supported by grants from the Netherlands Organization for Scientific Research NWO (Medical Research Council program grant GB-MW 940-38-011; ZonMW Brainpower grant 100-001-004; ZonMw Risk Behavior and Dependence grant 60-60600-97-118; ZonMw Culture and Health grant 261-98-710; Social Sciences Council medium-sized investment grants GB-MaGW 480-01-006 and GB-MaGW 480-07-001; Social Sciences Council project grants GB-MaGW 452-04-314 and GB-MaGW 452-06-004; NWO large-sized investment grant 175.010.2003.005; NWO Longitudinal Survey and Panel Funding 481-08-013); the Dutch Ministry of Justice (WODC), the European Science Foundation (EuroSTRESS project FP-006), Biobanking and Biomolecular Resources Research Infrastructure BBMRI-NL (CP 32), the participating universities, and Accare Center for Child and Adolescent Psychiatry. Statistical analyses were carried out on the Genetic Cluster Computer (http://www.geneticcluster.org), which is financially supported by the Netherlands Scientific Organization (NWO 480-05-003) along with a supplement from the Dutch Brain Foundation.

(TROMSO) University of Tromsø, Norwegian Research Council (project number 185764)

(Twingene) This project was supported by grants the US National Institutes of Health (AG028555, AG08724, AG04563, AG10175, AG08861), the Swedish Heart-Lung Foundation, the Swedish Foundation for Strategic Research, the Royal Swedish Academy of Science, and ENGAGE (within the European Union Seventh Framework Programme, HEALTH-F4-2007-201413), the Ministry for Higher Education, the Swedish Research Council (M-2005-1112 and 2009-2298), GenomEUtwin (EU/QLRT-2001-01254; QLG2-CT-2002-01254), NIH grant DK U01-066134, Knut and Alice Wallenberg Foundation (Wallenberg Academy Fellow), European Research Council (ERC Starting Grant), Swedish Diabetes Foundation (grant no. 2013-024), Swedish Research Council (grant no. 2012-1397), and Swedish Heart-Lung Foundation (20120197). The SNP Technology Platform is supported by Uppsala University, Uppsala University Hospital and the Swedish Research Council for Infrastructures. RJS is funded by the SRP diabetes programme at Karolinska Institutet. The computations were performed on resources provided by SNIC through Uppsala Multidisciplinary Center for Advanced Computational Science (UPPMAX) under Project b2011036.

(TWT2D) This project was supported by grants from the Academia Sinica, Taipei, Taiwan, and Ministry of Science and Technology (Nation N).

(WGHS) The WGHS is supported by the National Heart, Lung, and Blood Institute (HL043851 and HL080467) and the National Cancer Institute (CA047988 and UM1CA182913), with collaborative scientific support and funding for genotyping provided by Amgen.

(WHITEHALL II) The Whitehall II study has been supported by grants from the British Medical Research Council (MRC, K013351); the British Heart Foundation (PG/29605); and the National Heart, Lung, and Blood Institute (R01HL036310)
